# The effectiveness of optimal exercise-based strategy for patients with hip fracture: a systematic review and Bayesian network meta-analysis

**DOI:** 10.1038/s41598-023-37509-y

**Published:** 2023-06-29

**Authors:** Rong-jia Pan, Si-jie Gui, Yu-Lian He, Fang Nian, Xiao-Yan Ni, Yan-hui Zhou, Man-yi Wang, Jing-jing Wu, Gu-qing Zeng, Jing-hong Liang, Dan Peng

**Affiliations:** 1 School of Nursing, Hengyang Medical School, University of South China, 28 West Changsheng Road, Hengyang, 421001 Hunan People’s Republic of China; 2 Department of Orthopedics, The Second Xiangya Hospital of Central South University, Changsha, 410011 Hunan People’s Republic of China; 3 Department of Orthopedics and Trauma, the First Affiliated Hospital, Hengyang Medical School, University of South China, Hengyang, 421001 Hunan People’s Republic of China; 4 Department of Maternal and Child Health, School of Public Health, Sun Yat-Sen University, No.74 Zhongshan 2nd Road, Yuexiu District, Guangzhou, 510080 China

**Keywords:** Trauma, Fracture repair, Geriatrics, Public health, Quality of life, Genetics research, Disability

## Abstract

The implementation of exercise intervention (EI) presents a promising and economical way for patients with hip fracture. However, the optimal type of EI remains unclear. The objective of this study is to evaluate the efficacy of various EI approaches and identify the optimal intervention for improving the prognosis of patients with hip fracture. A comprehensive search of Medline (via PubMed), Web of Science, Embase, Cochrane Central Register of Controlled Trials, CINAHL, CNKI, Wan Fang, VIP, and CBM was conducted from their earliest records to June 2022. The included randomized controlled trials (RCTs) included at least one type of exercise for patients with hip fracture. The methodological quality of these trials was assessed using the Cochrane Collaboration Risk of Bias Tool. All direct and indirect comparisons were analyzed by Stata 14.0 and OpenBUGS 3.2.3 software. The primary outcome was hip function, and the secondary outcomes were activity of daily living (ADL), walking capacity and balance ability of patients. Based on the ranking probabilities, resistance exercise (RE) was ranked as the most effective among all exercise interventions (surface under cumulative ranking curve values [SUCRA]: 94.8%, [MD]: − 11.07, [Crl]: − 15.07 to − 7.08) in improving the efficacy of patients' hip function, followed by balance exercise (BE) ([SUCRA]:81.1%, [MD]: − 8.79, [Crl]: − 13.41 to − 4.18) and muscle strength exercise ([SUCRA]:57.6%, [MD]: − 5.35, [Crl]: − 9.70 to − 0.95). For the improvement of ADL for patients with hip fracture, BE ([SUCRA]:98.4%, [MD]: − 17.38, [Crl]: − 23.77 to − 11.04) may be the best EI. The findings of this study indicate that RE and BE might be the best approach to improve prognosis for patients with hip fracture. However, further rigorous and meticulously planned RCTs are required to substantiate the conclusions drawn from this study.

## Introduction

Hip fracture is a severe, debilitating condition with increasing prevalence globally^1^. The mortality rate within 2 years after hip fracture is 32.7%^2^ and the risk of refracture within 3 years increases by 30–40%^3^. Moreover, the patient's physical function and walking ability are significantly reduced, and the ability to self-care is lost after hip fracture, which seriously affects the patient's quality of life^4^. With the acceleration of population aging, the medical and financial burden associated with hip fracture will be a major challenge for society and families^5^. Therefore, it is crucial to improve the prognosis and activities of daily living (ADL) for patients with hip fracture.

There are several ways to promote recovery of patients after hip fracture, mainly including drug therapies, transcutaneous electric nerve stimulation (TENS) and exercise-based interventions (EIs). Nonetheless, drug therapies might have potential side effects, such as bisphosphonates may cause a significant decrease in bone density and increase the risk of secondary fractures when taken continuously for 3–5 years^6^. Overdose of vitamin D might cause kidney stones or cardiovascular accidents, affecting the life of the patient^7^. TENS is a new non-invasive acupuncture treatment^8^, however, it is limited in terms of who can implement it and where it can be used, and it lacks home applicability and population universality. Furthermore, the scientific rationale and criteria for the selection of acupuncture points, frequency, and duration of stimulation remain unclear, inappropriate manipulations may diminish therapeutic effect^9^. EI mainly includes aerobic exercise (AE), resistance exercise (RE), muscle strength exercise (MSE), balance exercise (BE), and weight-bearing exercise (WBE)^10,11^. Previous studies proposed that EIs might have a positive effect on physical function in patients with hip fracture^12,13^. Experimental studies^14^ have suggested that EIs could maintain high peroxisome proliferator-activated receptor-γ coactivator-1α levels, and inhibit in vivo Forkhead boxO3 induction, which reduces muscle atrophy, maintains muscle function and increases hip stability. More importantly, exercise as a promising treatment for patients with hip fracture was recommended by guidelines^15^. Consequently, EIs have been widely applied to patients with hip fracture due to its high safety and well-proven efficacy with few side effects.

Although previous research has shown the prognostic value of EIs in patients with hip fracture, the evidence regarding the effectiveness of EIs remains fragmented and controversial. Firstly, studies have shown that EIs could improve ADL and walking ability of patients^16^, but Magaziner^17^ suggested that the improvement of ADL and walking ability of patients with hip fracture was not statistically significant after EIs. Due to some discrepancies in the results of previous RCTs, a high-quality meta-analyse is urgently needed to summarize the current clinical evidence. Secondly, there are many limitations to these studies, including single-center recruitment, small sample sizes, and limited follow-up times^12,18^, which to some extent affect their generalizability and accuracy for judging the efficacy of EIs, and their long-term effects still need to be confirmed. Thirdly, the single outcome indicator in the previous literature only reflects the efficacy of EI with hip fracture patients in a one-sided way^19^, which affects the medical staff to make an overall and comprehensive judgment on the efficacy of EIs and cannot meet the actual clinical needs. Finally, the optimal exercise type for patients with hip fracture is unclear as traditional meta-analyse mainly focus on comparisons of single EI^20^ and lacks direct comparisons of different EIs, it is difficult for healthcare professionals to develop the most effective exercise rehabilitation programs. Besides, in recent years, much literature has been published on EIs after hip fracture^21,22^, we urgently need to update the evidence to verify the effectiveness of the best interventions.

Network meta-analysis (NMA) is a new method for comparing direct and indirect evidence that helps researchers gather evidence from multiple RCTs and compares the relative effectiveness of multiple interventions^23^. It overcomes the limitation of traditional pare-wise meta-analysis and ranks the probability of each intervention’s relative efficacy^24^. Our study aims to identify the optimal exercise-based strategy through a Bayesian NMA and provides a reference for policymakers and clinical researchers.

## Materials and methods

The Preferred Reporting Items for Systematic Reviews and Meta Analyses-Network Meta-Analyses (PRISMA-NMA) guidelines^25^ were followed when conducting and reporting our NMA (Supplement File S1). This study has been registered on PROSPERO (CRD4202022340737).

### Search strategies

A systematic literature search was carried out in Medline (via PubMed), Excerpta Medica Database (Embase), Cochrane Central Register of Controlled Trials (CENTRAL), China National Knowledge Infrastructure database (CNKI), Web of Science, Wan Fang database, Cumulative Index to Nursing and Allied Health Literature (CINAHL), China Science and Technology Journal Database and China Biomedical Literature Database (CBM) until June 23rd, 2022. The search was not restricted by language and publication date. We used combined terms medical subject headings and text words around “hip fractures”, “resistance training”, “aerobic exercise”, “postural balance”, “muscle strength”, “weight-bearing exercise” and “randomized controlled trial” to search for relevant studies. In addition, in order to ensure the comprehensiveness of the search results, we manually searched the references of published meta-analyses and grey literature, such as conference proceedings and academic degree dissertations. The full search strategies were available in Supplement File S2.

### Inclusion criteria and literature screening

According to the PICOS guidelines, we conducted a literature screening. The detailed inclusion criteria were as follows: (1) participants were diagnosed with hip fracture according to X-ray and computed tomography (CT) scan^26^; (2) the intervention group include anyone type of exercise intervention (such as AE, RE, BE); (3) the control group received usual care and did not receive a structured exercise intervention; (4) at least included one of the specified outcomes (hip function, ADL, walking capacity, balance ability); (5) study design is RCT. Exclusion criteria were as follow: (1) participants were complicated with other diseases, such as dementia; (2) mixed different interventions were reported in literature; (3) raw data were incomplete or could not be acquired in the literature; (4) study design was non-randomized clinical trials, case reports, reviews or protocols. The first step was to eliminate duplication using Endnote X9 software. Afterward, two researchers independently selected references by reading the titles and abstracts of references. Finally, we further screened the full texts of relevant studies. The disagreement was resolved by the third reviewer.

### Data extraction and outcomes measurement

Two investigators independently extracted the data and cross-checked the results. The following data were extracted from each article: (1) title, journal, first author, year of publication; (2) age, gender and baseline condition of included participants; (3) sample size, intervention type, intervention duration, region of included studies; (4) the primary outcome and secondary outcomes for all the included studies. We have extracted data including mean values and standard deviation or standard error of the mean of inclusion studies. In the event of uncertainty regarding crucial information and data in included studies, the original researcher was contacted via email to procure necessary data for this study.

The hip function of the patient was assessed as a primary outcome by the Harris hip scoring scale. Secondary outcomes included ADL, walking capacity and balance ability of the participants. ADL was measured by the Barthel index or ADL scale. Walking ability was measured by a six-minute walk test. Berg Balance Scale was used to measure balance ability.

### Risk of bias and quality assessment

Two researchers independently assessed the risk of bias (ROB) for all included studies according to the Cochrane Collaboration’s risk of bias tool^27^. These studies were graded as having low, high or unclear ROB based on the following items: random sequence generation, allocation concealment, blinding of participants, blinding of the outcome assessor, incomplete data, selective reporting, and other sources of bias.

### Data analyses

The I^2^ statistic was used to determine the degree of heterogeneity between studies, with values of 25%, 50%, and 75% respectively representing low, moderate, and high heterogeneity^28^, respectively. We first judged whether the measurement tools were consistent between studies, then we presented continuous outcomes using weighted mean differences (WMD) or standardized mean differences (SMD) with 95% confidence interval. If the standard deviation (SD) is missing,we estimate missing SD by standard errors, confidence interval, t-value, and *P*-value for single or combined conversion^29,30^.

Transitivity assumption could further impact the validity of NMA^31^. We considered transitivity by assessing clinical and methodological comparability, such as subjects and study design. Parameters were assessed using maximum likelihood and Bayesian inference for indirect efficacy comparisons, we conducted NMA based on hierarchical Bayesian models to compare the effects of different EIs. The direct and indirect comparison results in a network diagram were presented using the method of multivariate meta-analysis^32^. Three Markov chains were initialized to assess convergence^33^, then the chain for 50,000 iterations was run and the first 20,000 Markov Chain Monte Carlo iterations were discarded as burn in^34^. Moreover, we assessed convergence of each parameter using trace history and Brooks–Gelman–Rubin diagnostic plots^35^. Besides, we compared the efficacy of various EIs in patients with hip fracture, and the effect size of each EI will be expressed as MD and 95% credible interval (95%CrI), we ranked the EIs effects according to the cumulative ranking probability curve (surface under the cumulative ranking area, SUCRA). A larger SUCRA value implies more effective interventions because SUCRA values reflect an intervention's effectiveness^36^. We assessed inconsistency by fitting both an inconsistency model and a consistency model. Node-splitting method was used to assess whether direct and indirect evidence on a specific node was in agreement^37^. If the difference was not statistically significant (*p* > 0.05), it indicated that the result of direct comparison and indirect comparison was consistent^38^. Publication bias were identified with funnel plots, whereby asymmetries in the funnel plot indicated publication bias^39^. All analyses were performed using Stata version 14.0 (StataCorp, College Station, TX, USA) and OpenBUGS version 3.2.3.

## Results

### Baseline selection

**Figure 1:**
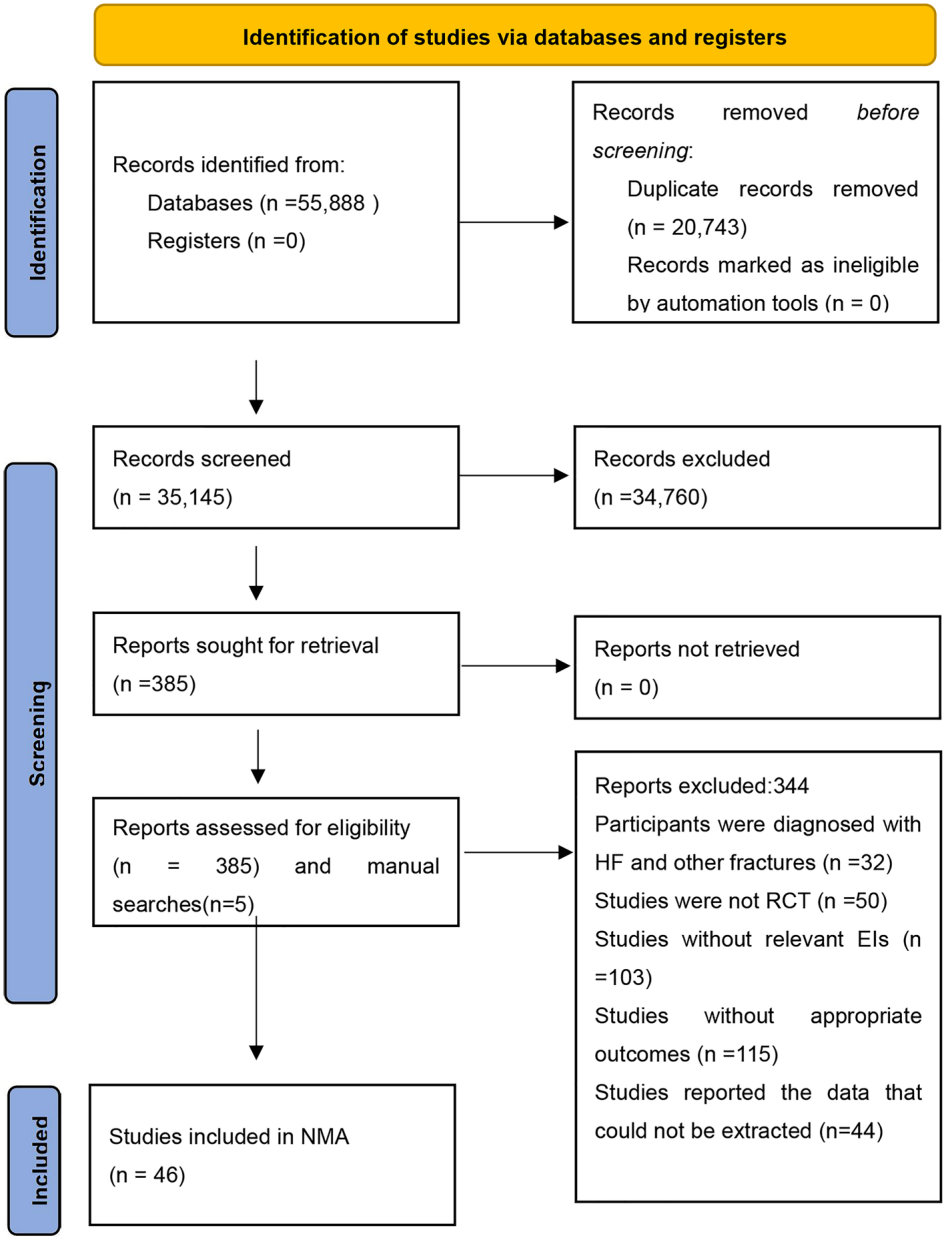
Selection of studies for inclusion. *EI* exercise-based intervention, *HF* hip fracture, *NMA* network meta-analysis, *RCT* randomized controlled trial.

A total of 55,888 articles were identified after an initial database search and 20,743 studies were removed after the removal of duplicates. After the screening of titles and abstracts, 34,760 studies did not meet the eligibility criteria, afterward, a total of 390 articles were selected for full-text screening, 5 of which were from manual searches. After the full-text screening, 344 records were excluded due to various reasons: 32 studies enrolled the participants who were diagnosed with multiple fractures, 50 studies were not RCTs, 103 studies without including EIs that we defined in this study, 115 studies lacked appropriate outcomes, 44 studies reported the data that could not be extracted. Finally, 46 studies were included for NMA (Supplement File S3). The flow diagram of the PRISMA screening process was provided in Fig. 1.

### Characteristics of included trials

Characteristics of the included studies in this NMA were presented in Table 1. Forty-six studies included 3,286 participants aged between 52 to 85 years and the data were published between 1997 and 2022. Moreover, participants were mostly female (56.9%) and most of the interventions lasted from 12 to 24 weeks. Regarding the regions where the research was carried out, 4 studies were conducted in the USA, 12 studies in Europe, 27 studies were in Asia and the remaining 3 in other regions.

**Table 1:** The characteristics of studies included in the NMA.

Study, year	Participants (exercise *vs* control)					Interventions		Outcomes
	Type	Sample size	Age (EG)(${\overline{\text{x}}}$ ± s)	Age (CG)(${\overline{\text{x}}}$ ± s)	Gender (Female/male)	Duration (week)	Region	
Sherrington et al., 1997	WBE	21 *vs* 21	80.00 ± 8.10	77.10 ± 8.20	8/13 *vs *1/20	4	Australia	①
Hauer et al., 2002	RE	15 *vs* 13	81.70 ± 7.60	80.80 ± 7.00	NR	12	Germany	①②③④
Sherrington et al., 2003	WBE	41 *vs* 39	81.00 ± 7.00	81.10 ± 8.30	14/27 *vs* 12/27	2	Australia	①②③
Binderet al., 2004	RE	46 *vs* 44	80.00 ± 7.00	81.00 ± 8.00	13/33 *vs* 10/34	24	USA	①②③④
Suetta et al., 2004	RE	13 *vs* 12	69.00	68.00	7/6 *vs* 7/5	12	Denmark	①②
Sherrington et al., 2004	WBE	40 *vs* 40	80.10 ± 7.50	77.20 ± 8.90	10/30 vs 6/34	4	Australia	①②③
Mangione et al., 2005	AE vs RE	12 *vs* 11*vs*10	79.80 ± 5.60/77.90 ± 7.90	77.80 ± 7.30	3/9/4 *vs *2/8/7	12	USA	①③
Mard et al., 2008	MSE	23 *vs* 20	74.00 ± 6.00	74.00 ± 7.00	8/16 *vs* 6/16	12	Finland	①
Mendelsohn et al., 2008	AE	10 *vs* 10	80.30 ± 7.40	81.10 ± 7.20	3/7 *vs* 3/7	4	USA	①②④
Portegijs et al., 2008	RE	23 *vs* 20	73.80 ± 6.60	74.10 ± 7.20	NR	12	Finland	①②
Mangione et al., 2010	RE	14 *vs* 12	79.60 ± 5.90	82.00 ± 6.00	2/12 *vs* 3/9	10	USA	①③
Kui et al., 2011	WBE	78 *vs* 74	71.80 ± 3.70	72.30 ± 3.70	30/48 *vs* 23/51	12	China	④
Yan et al., 2012	BE	20 *vs* 20	62.70 ± 6.30	61.34 ± 5.20	7/13 *vs* 9/11	24	China	③
Singh et al., 2012	RE	62 *vs* 62	80.10 ± 10.10	78.40 ± 9.00	19/43 *vs* 20/42	48	Australia	③
Sylliaas et al., 2012	RE	48 *vs* 47	82.40 ± 6.50	82.20 ± 5.10	9/39 *vs* 10/38	12	Norway	①②③
Morishima et al., 2014	AE	14 *vs* 14	60.30 ± 7.40	59.90 ± 5.40	0/14 *vs* 0/14	12	Japan	①
van Ooijen et al., 2016	AE	24 *vs* 23	82.90 ± 6.50	83.30 ± 8.00	8/16 *vs* 2/21	6	Netherlands	①③
Zhao et al., 2016	AE	60 *vs* 60	70.69 ± 8.76	70.15 ± 8.62	31/29 *vs* 32/28	12	China	③④
Xing et al., 2016	AE	50 *vs* 50	70.69 ± 8.76	70.15 ± 8.62	24/26 *vs* 24/26	12	China	③④
Zhang et al., 2017	MSE	30 *vs* 30	67.43 ± 2.81	68.27 ± 3.38	18/12 *vs* 16/14	2	China	③④
Xu et al., 2017	BE	35 *vs* 35	63.43 ± 7.20	64.20 ± 8.48	21/14 *vs* 20/15	24	China	②④
Monticone et al., 2018	RE	26 *vs* 26	77.20 ± 6.60	77.70 ± 7.50	7/19 *vs* 8/18	3	Italy	②③④
Wang et al., 2019	RE	53 *vs* 53	67.20 ± 2.10	66.10 ± 2.50	22/31 *vs* 21/32	12	China	③④
Kang et al., 2019	AE	34 *vs* 34	57.19 ± 10.08	55.71 ± 10.91	21/13 *vs* 20/14	48	China	③④
Wu et al., 2019	AE	50 *vs* 50	71.64 ± 5.59	71.42 ± 7.20	16/34 *vs *17/33	72	China	③④
Dong et al., 2019	AE	46 *vs* 46	60.34 ± 2.19	60.78 ± 2.23	38/8 *vs* 37/9	12	China	③④
Stasi et al., 2019	AE	48 *vs* 48	77.50 ± 4.00	77.50 ± 4.50	12/36 *vs* 12/36	12	Greece	①
Cai et al., 2020	MSE	49 *vs* 49	66.53 ± 5.71	65.71 ± 6.32	19/30 *vs* 20/29	12	China	①③④
Xu et al., 2020	BE	42 *vs* 41	67.26 ± 3.29	67.58 ± 3.61	25/17 *vs* 23/18	24	China	②④
Qin et al., 2020	RE	43 *vs* 37	67.77 ± 3.22	68.34 ± 3.05	22/21 *vs* 18/19	12	China	③④
Oh et al., 2020	RE	19 *vs* 19	76.94 ± 9.43	81.15 ± 4.90	6/13 *vs* 6/13	1.5	Korea	②③④
Kim et al., 2020	AE	17 *vs* 17	52.82 ± 5.96	51.82 ± 5.91	13/4 *vs *13/4	4	Korea	④
Wang et al., 2020	WBE	41 *vs* 41	51.50 ± 1.40	49.50 ± 1.80	25/16 *vs* 27/14	12	China	③④
Chi et al., 2020	WBE	38 *vs* 38	69.83 ± 4.12	70.15 ± 3.82	23/15 *vs* 24/14	12	China	③④
Sun et al., 2020	MSE	47 *vs* 43	68.73 ± 6.92	68.07 ± 7.01	25/22 *vs* 22/21	16	China	③④
Li et al., 2020	MSE	40 *vs* 40	64.77 ± 8.21	64.65 ± 8.65	21/19 *vs *23/17	12	China	③④
Xu et al., 2021	RE	49 *vs* 49	72.34 ± 6.23	73.18 ± 6.82	29/20 *vs* 26/23	8	China	③④
Liu et al., 2021	BE	63 *vs* 62	64.71 ± 5.19	63.85 ± 5.43	28/35 *vs* 27/35	24	China	②④
Guo et al., 2021	BE	33 *vs* 33	68.54 ± 3.01	68.56 ± 3.02	16/17 *vs* 15/18	4	China	②③④
Ding et al., 2021	AE	40 *vs* 40	69.87 ± 8.81	70.21 ± 8.60	22/18 *vs* 21/19	12	China	③④
Wang et al., 2021	AE	49 *vs* 49	70.63 ± 6.96	70.05 ± 6.52	35/14 *vs* 31/18	12	China	③④
Corna et al., 2021	WBE	20 *vs* 20	83.60 ± 6.70	85.70 ± 8.40	5/15 *vs* 5/15	3	Italy	④
Paulsson et al., 2021	WBE	11 *vs* 18	79.20 ± 9.00	81.30 ± 8.00	1/10 *vs* 3/15	8	Sweden	③④
Overgaard et al., 2022	MSE	50 *vs* 50	78.30 ± 7.90	75.70 ± 8.10	6/44 *vs* 13/37	12	Denmark	①②③
Yan et al., 2022	MSE	50 *vs* 50	68.87 ± 6.27	67.19 ± 6.87	27/23 *vs *24/26	9	China	③④
Li et al., 2022	RE	20 *vs* 20	54.08 ± 2.73	52.18 ± 3.01	15/5 *vs* 12/8	4	China	③④

*AE* aerobic exercise, *BE* balance exercise, *CG* control group, *EG* experimental group, *MSE* muscle strength exercise, *NR* not reported, *RE* resistance exercise, *WBE* weight-bearing exercise; ①, walking capacity;②, balance ability;③, activity of daily living;④, hip function.

### Network meta-analysis

**Figure 2:**
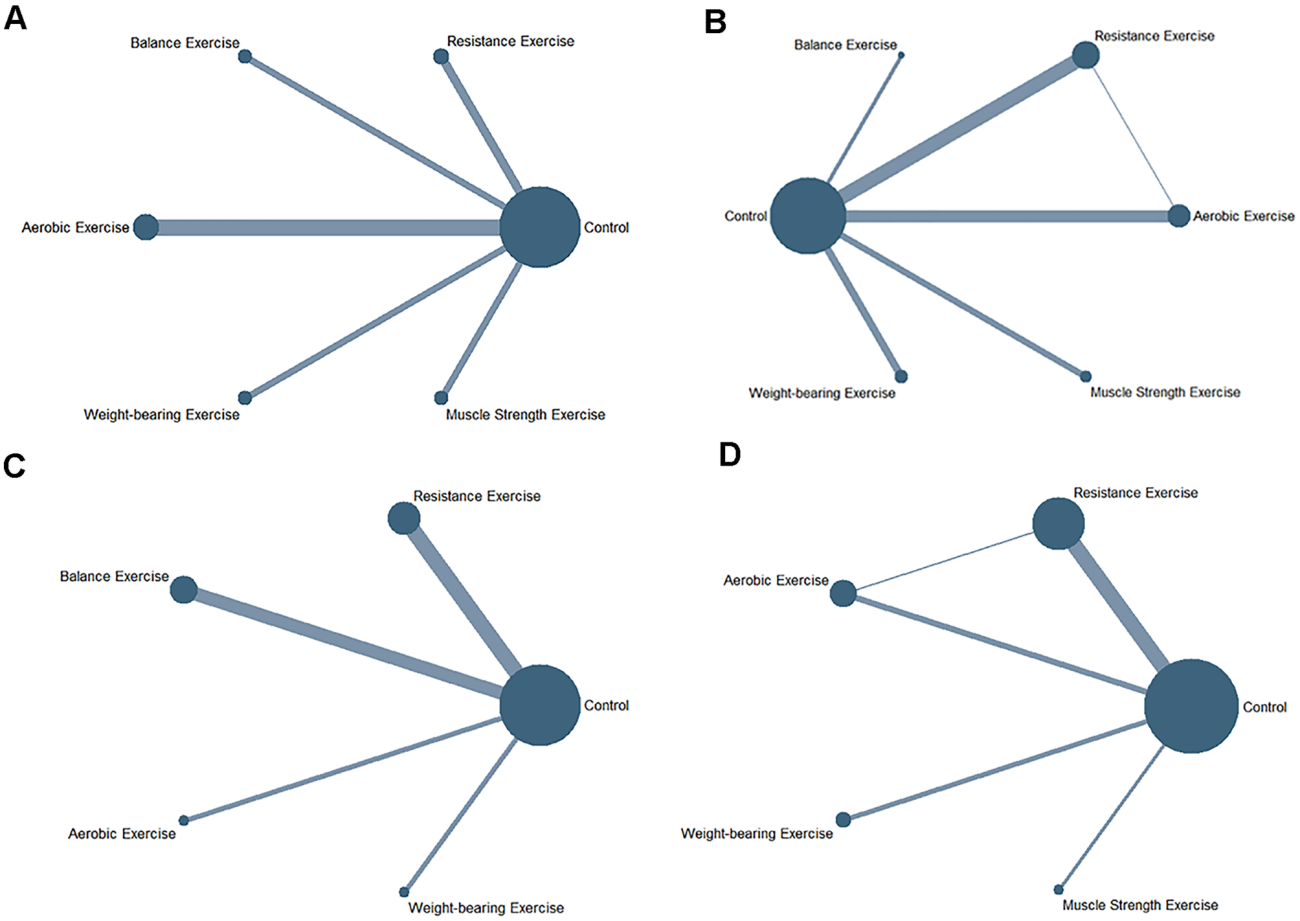
Network meta-analysis of eligible comparisons for hip function (**A**), activities of daily living (**B**), walking capacity (**C**), and balance ability (**D**).

Evidence networks about four outcome indicators were shown in Fig. 2. One of the evidence networks was a three-arm study, the others were two-arm studies. The size of the dots represented different EIs and the lines connecting them represented the direct comparison of EIs.

### Primary outcome and secondary outcome

The primary clinical outcome for our study was hip function, 31 studies (2395 participants) were included. RE (MD = − 11.07, 95%CrI: − 15.07, − 7.08), BE (MD = − 8.79, 95%CrI: − 13.41, − 4.18), MSE (MD = − 5.35, 95%CrI: − 9.70, − 0.95) showed statistically significant benefits compared with control group (CG). Besides, RE (MD = − 8.86, 95%CrI: − 15.01, − 2.71) (MD = − 9.09, 95%CrI: − 14.20, − 3.98) were superior to WBE and AE. BE (MD = − 6.58, 95%CrI: − 13.12, − 0.02) (MD = − 6.81, 95%CrI: − 12.42, − 1.22) was significantly better than WBE and AE. Based on the effectiveness of prognosis for patients with hip fracture, we employed SUCRA to rank five interventions: RE (94.8%) and BE (81.1%) were significantly effective, MSE (57.6%) and WBE (31.3%) had relatively low efficacy, AE (29.5%) was efficacy while CG (5.9%) (Table 2 and Supplement Fig. S1). No obvious heterogeneity was detected for hip function (SD = 0.1987). The test of global inconsistency did not show any significant difference for the hip function (*p*-value = 0.5572). The funnel plots did not show symmetric distribution, indicating a hint of publication bias (Supplement Fig. S2).

**Table 2:**
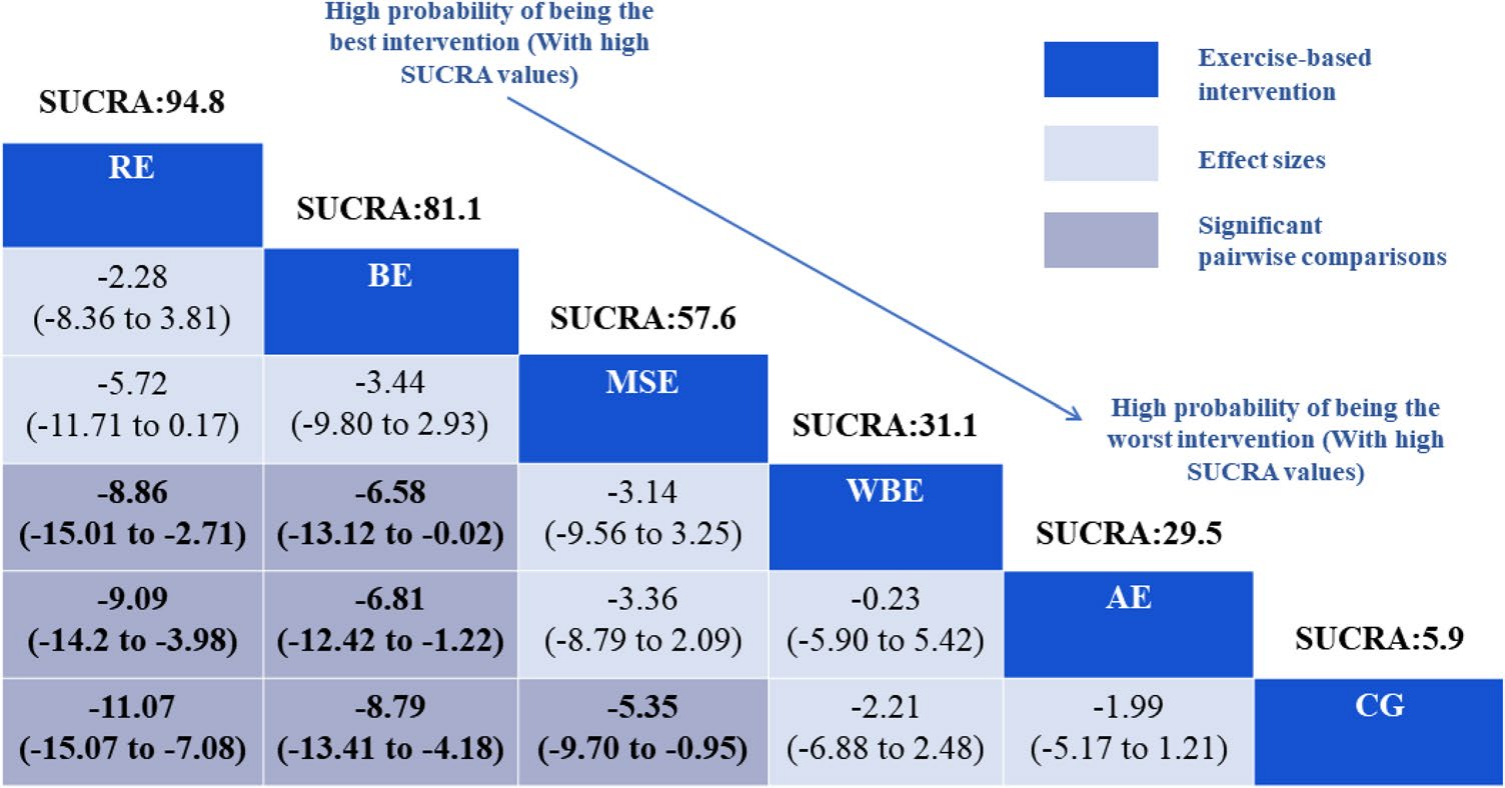
Relative effect sizes of different exercise interventions’ efficacy based on hip function.

Comparative effectiveness results for hip function. Each cell shows an MD with a 95%CrI. Some numbers at the top of boxes are SUCRA values. The dark blue boxes indicate the type of campaign intervention, blue grey boxes represent significant pairwise comparisons were highlighted.

*AE* aerobic exercise, *BE* balance exercise, *CG* control group, *Crl* credibility interval, *MD* mean difference, *MSE* muscle strength exercise, *RE* resistance exercise, *SUCRA* surface under cumulative ranking curve values, *WBE* weight-bearing exercise.

The secondary outcomes of this study were ADL, walking capacity and balance ability. For ADL, 33 studies (2,496 participants) were included. BE (MD = − 17.38, 95%CrI: − 23.77, − 11.04), WBE (MD = − 9.66, 95%CrI: − 13.99, − 5.32), RE (MD = − 7.73, 95%CrI: − 10.88, − 4.58), MSE (MD = − 4.96, 95%CrI: − 9.27, − 0.67), AE (MD = − 4.64, 95%CrI: − 8.23, − 1.04) were significant than the CG. In addition, BE was significantly superior to WBE, RE, MSE, AE in improving patients’ ADL. The SUCRA analysis indicated that BE (98.4%) had the highest probability of being the most effective EI, followed by, WBE (73.4%), RE (59.8%), MSE (35.2%), AE (31.8%). (Supplement Fig. S3 and Table S1) No obvious heterogeneity was detected for ADL (SD = 0.1865). The results of the inconsistency test (inconsistency factor [IF] = 0.09, 95% confidence interval[95%CI]:0.00 to 4.06) showed no inconsistency for the outcome of ADL (Supplement Fig. S4). The examination results of the funnel plot for ADL indicated that there was publication bias. (Supplement Fig. S5).

The remaining two outcome indicators for the EIs were walking capacity and balance ability. RE (MD = − 4.97, 95%CrI: − 8.51, − 1.44) could significantly improve the walking capacity of patients compared with that of CG. Moreover, the study results indicated that BE (MD = − 7.98, 95%CrI: − 11.77, − 4.23) and RE (MD = − 5.87, 95%CrI: − 9.27, − 2.44) were more effective than CG in improving walking capacity and balance ability (Supplement Tables S2 and S3). The SUCRA values resulting in top-ranked classes for walking capacity were RE (81.9%) and for balance ability were BE (88.4%) (Supplement Fig. S6). For walking capacity, the result of the inconsistency test (IF = 0.22, 95%CI 0.00 to 2.32) was shown in Supplement Fig. S7. No obvious heterogeneity was detected for walking capacity (SD = 0.1661). For balance ability, no inconsistency was found through global and loop-specific approach (p-value = 0.8547). Mild heterogeneity was detected for balance ability (SD = 0.5814). A comparison-adjusted funnel plot of walking capacity and balance ability were displayed in Supplement Fig. S8, indicating the existence of publication bias.

### Quality of the included studies

Details of the ROB assessments were presented in Supplement Figs. S9 and S10. Thirty-nine studies described the methods for random sequence generation in detail and only 19 studies described the allocation concealment. Blinding was designed for participants and investigators in 14 studies and outcome assessors in 16 studies. The outcomes of 41 studies were largely complete, and detailed descriptions were made of the rates and reasons for loss to follow-up. Thirty-four studies reported all prespecified (primary and secondary) outcomes in detail. In the 39 studies, the baseline data of the intervention group and the control group were comparable.

## Discussion

This NMA confirmed that EIs were beneficial for hip fracture patients' hip function, ADL, walking capacity and balance ability. Furthermore, we found that RE and BE were the most effective rehabilitation exercises for functional recovery and capacity improvement after surgery.

In our study, RE (SUCRA: 94.8%) and BE (SUCRA: 81.1%) were beneficial to hip function of patients. In comparison with CG in included studies, BE (SUCRA: 88.4%) and RE (SUCRA: 66.1%) were more effective in improving balance ability of patients. Meta-analysis showed that RE could improve physical function, balance, lower-limb strength in patients with hip fracture^40,41^, which is consistent with the results of our study. Hermann^42^ also reported that progressive explosive-type RTs could significantly reduce exercise-related pain and increase leg muscle power, thus maintain body balance. Although the potential mechanism for RE improving balance ability and hip function is unclear, some findings have shown that RE activated the PI3K/Akt/mTOR and AMPK/Sirt1 signaling pathways and inhibited the NFκB/NLRP3/IL-1β signaling pathway, in addition, it also increased muscle oxygen consumption, induced muscle protein synthesis, and increased skeletal muscle mass and function^43^. Moreover, RE also downregulated the autophagy-specific protein LC31/LC3-1 ratio, reduced p62 protein levels and increased autophagy-regulating proteins such as Beclin1, ATG5\12\7, improved myocyte autophagy impairment and helped to maintain skeletal muscle strength and muscle mass^44^. Adequate muscle and bone mass is important for maintaining hip function and balance in postural maintenance and dynamic daily activities, several mechanisms explained that RE had a beneficial effect on patients with hip fracture. In addition, RE of the muscles around the hip can be effective in improving hip function by gradually increasing joint mobility, increasing the amount of movement and preventing joint adhesions^45^. BE aims to maintain gait stability and prevent fall occurrence, including multiple types of balance exercises, such as single-leg standing balance, balance capacity after sudden perturbation, and postural control^46^. Unipedal standing BE was applied to 527 elderly patients^47^, which demonstrated the effectiveness of preventing falls and improving balance ability. Our results also agreed well with those of a previous study by Lima^48^. BE could strengthen the coordination and balance after hip fracture by enhancing erector spinae and gluteus medius muscle activity^49^. Furthermore, BE could reduce overactive proprioceptive feedback and restore vestibular orientation in patients^50^, which helped to prevent the occurrence of falls and maintain balance. Aging could cause joint stiffness and impair muscle strength, which are all risk factors for balance^51^. The implementation of BE may result in heightened muscle strength, notable advancements in the patient's capacity to shift their center of gravity, improved postural stability and balance, expedited functional recuperation of the lower extremities, and enhanced mobility of the hip joint and hip function.

Based on the cumulative ranking results, BE (SUCRA: 98.4%) and WBE (SUCRA: 73.4%) were superior to CG in improving ADL of patients. BE is an inexpensive and effective treatment means for hip fracture, its effectiveness in improving ADL is gradually being proved^52^, which is in agreement with our study results. This may be related to the fact that BE enables the patient to gradually perform bed-chair transfers and sit-to-stand transfers independently, thereby improving the patient's trunk control and ADL^53^. WBE has been regarded as the principal physical activity for promoting bone health^54^. The findings of our study indicate that WBE rank second in terms of effectiveness in enhancing ADL among patients, after BE. Warren^55^ compared WBE group with the non-weight-bearing group found that early WBE could improve physical fitness and mobility, and directly facilitate the improvement of ADL. This may be early WBE can stimulate the proliferation and differentiation of osteoblasts, accelerate bone tissue growth, promote functional recovery, and thus improve ADL^56,57^. However, in the affected limb, excessive WBE might lead to osteonecrosis or delayed healing. Therefore, there is a lack of consensus on WBE standards, and relevant research should be strengthened in the future to fill this gap.

The results of SUCRA showed that RE and MSE have better efficacy in improving walking capacity for patients with hip fracture. It might relate to the mechanism that RE activates Akt activity and FGF21 gene, reduces skeletal muscle decay, maintains joint mobility and improves walking capacity^58^. MSE for patients recovering from hip fracture may be necessary to further reduce skeletal muscle inflammation and improve muscle function. MSE is based on a correct biomechanical and kinematic analysis to train the patient in muscle strength and hip stability. Mitchell^59^ implemented quadriceps’ strength training, the result suggested that the knee strength of the patients in the trained group significantly increased (157%) during quadriceps extension compared with that in the untrained group (63%). Furthermore, a systematic review^60^ has shown that quadriceps training programmers can improve leg extensor power and walking efficiency. The possible mechanism was that MSE could promote the proliferation of activated satellite cells and produce new myocytes^61^. Therefore, MSE could improve muscle strength and coordination, restore the greater walking ability to the patient. Furthermore, patients were prone to sedentary behavior after hip fracture, which would cause a more pronounced decrease in the number and strength of muscle fibers, and lead to muscle atrophy^62^, targeted muscle training might prevent symptoms from getting worse. However, the RCT literature included in this study had a short follow-up period, further high‐quality and long-term follow-up RCTs were recommended to examine long‐term effectiveness and benefits for patients with hip fracture.

With the aging of the population and the extension of life expectancy, the incidence of hip fracture is increasing. EIs have become a crucial means of enhancing hip function and ADL in hip fracture patients. This research has discovered that RE and BE are the most effective EIs for improving hip function and ADL in hip fracture patients, with broad applicability, cost-effectiveness, and comprehensiveness. Besides, they are suitable for implementation in both in-hospital and home-based rehabilitation settings. Therefore, this study provides novel evidence for hip fracture rehabilitation and holds significant reference value for promoting patient recovery.

### Strengths and limitations

The present study has the following strengths. First, our study is the first time to use NMA to verify the effectiveness of different EIs after hip fracture, and made up for the inadequacies of traditional meta-analysis in indirectly comparing multiple interventions. The language and publication date were not restricted in our study, our literature search strategy used multiple databases to identify as many studies as possible, the sample size is large and representative.

The limitations of our study should be discussed. A key limitation is the findings of our NMA that might have been influenced by large heterogeneity reported from different included studies, for example, some of them lacked the blindness in participation, intervenors or outcome assessors. Additionally, there was no standardized definition for nursing measures for the control group, in the included literature, some controls were given usual care and some literature controls were given usual physical activity, which led to uncertainty in the results of the study. Ultimately, the elderly population represents a demographic with a heightened likelihood of experiencing hip fractures. However, upon conducting a literature search, it was discovered that individuals within the age range of 50 to 60 years were at increased risk for hip fracture. Given the wide dissemination of the results, we incorporated this population into our study, not solely consisting of the elderly population.

Given the rapidly expanding population of older adults worldwide and the potential advantages of EI, including its cost-effectiveness, safety considerations and potential benefits, it is imperative to refine EI programs and conduct high-quality EIs for patients with hip fracture in the future research. We believe that our study provides evidence with rehabilitation success of patients with hip fracture. As an illustration, a study published subsequent to our literature search period had highlighted the effectiveness of EIs, gait and muscle strength were improved significantly for patients with hip fracture after 16-week intervention^63^. Hence, our research has the potential for fueling subsequent developments and research in the field of hip fracture rehabilitation, it is expected to be widely used in orthopaedic rehabilitation field and provides novel evidence and reference values. The results of this study help healthcare practitioners to formulate optimal rehabilitation protocols for patients with hip fractures, which promotes optimal care for patients with hip fractures.

## Conclusions and implications

In summary, our study might provide strong evidence about RE as the optimal intervention in improving hip function for patients with hip fracture and offers implications for future studies. In addition, we have demonstrated that BE can improve ADL and balance for patients. However, due to insufficient literature numbers and moderate quality of studies, the results should be interpreted cautiously. Among the multitudinous exercise-based strategies, RE and BE as two of the most promising perspectives should continue to be explored and applied.

## Supplementary Information

Supplementary Information.

## Data Availability

All other data is available in the Supplementary Information files. Any further information is available upon request from the corresponding author.
