# Supplementary material for: The effectiveness of optimal exercise-based strategy for patients with hip fracture: a systematic review and Bayesian network meta-analysis
Source: Sci Rep. 2023 Jun 29;13:10521. doi: 10.1038/s41598-023-37509-y (PMC10310779; doi:10.1038/s41598-023-37509-y)
Supplement: Supplementary file 1 — Supplementary Information. [file 41598_2023_37509_MOESM1_ESM.docx]

**The effectiveness of** **optimal exercise-based strategy for elderly patients with hip fracture: A systematic review and Bayesian network**

**meta-analysis**

**Rong-jia Pan^1^**+**, Si-jie Gui^2^**+**, Yu-Lian He^3^**+**, Fang Nian^1^, Xiao-Yan Ni^2^, Yan-hui Zhou^3^, Man-yi Wang^1^, Jing-jing Wu^1^, Gu-qing Zeng^1^*, Jing-hong Liang^4*^, Dan Peng^2*^**

^1^ School of Nursing, Hengyang Medical School, University of South China, Hengyang, Hunan, P.R. China,421001

^2^ Department of Orthopedics, The Second Xiangya Hospital of Central South University, Changsha, Hunan, P.R. China,410011;

^3^ Department of orthopedics and trauma, the First Affiliated Hospital, Hengyang Medical School, University of South China, Hengyang, Hunan, P.R. China,421001;

^4^ Department of Maternal and Child Health, School of Public Health, Sun Yat-sen University, Guangzhou P. R. China,510080.

+These authors contributed equally to this work.

Correspondence to: Gu-qing Zeng, Jing-hong Liang, Dan Peng

**List of contents**

| **Title** | **Page** |
| --- | --- |
| **Supplemental** **File 1** PRISMA network meta-analysis checklist. | 3 |
| **Supplemental File 2** Search strategy. | 9 |
| **Supplemental File 3** References for included studies. | 19 |
| **Supplemental Figure 1** Plots of the surface under the cumulative ranking curves (SUCRA) for all comparisons in the hip function. | 25 |
| **Supplemental Figure 2** Funnel plots for the effects of exercise interventions on hip function. | 26 |
| **Supplemental Figure 3** Plots of the surface under the cumulative ranking curves (SUCRA) for all comparisons in the ADL. | 27 |
| **Supplemental Figure 4** Inconsistency plot for the ADL, assuming loop-specific heterogeneity estimates. | 28 |
| **Supplemental Figure 5** Funnel plots for the effects of exercise interventions on ADL. | 29 |
| **Supplemental Figure 6** Plots of the surface under the cumulative ranking curves (SUCRA) for all comparisons in walking capacity and balance ability. | 30 |
| **Supplemental Figure 7** Inconsistency plot for the walking capacity network, assuming loop-specific heterogeneity estimates. | 31 |
| **Supplemental Figure 8** Funnel plots for the effects of exercise interventions on walking capacity and balance ability. | 32 |
| **Supplemental Figure 9** Risk of bias graph. | 33 |
| **Supplemental Figure 10** Risk of bias summary. | 34 |
| **Supplemental Table 1** Relative effect sizes of different exercise interventions’ efficacy based on ADL. | 35 |
| **Supplemental Table 2** Relative effect sizes of different exercise interventions’ efficacy based on walking capacity. | 36 |
| **Supplemental Table 3** Relative effect sizes of different exercise interventions’ efficacy based on balance ability. | 37 |

**Supplemental File 1 PRISMA NMA Checklist of Items to Include When Reporting A Systematic Review Involving a Network Meta-analysis**

| **Section/Topic** | **Item #** | **Checklist Item** | **Reported on Page #** |
| --- | --- | --- | --- |
| **TITLE** |  |  |  |
| Title | 1 | Identify the report as a systematic review *incorporating a network meta-analysis (or related form of meta-analysis).* | ***Page1*** |
|  |  |  |  |
| **ABSTRACT** |  |  |  |
| Structured summary | 2 | Provide a structured summary including, as applicable:  **Background:** main objectives  **Methods:** data sources; study eligibility criteria, participants, and interventions; study appraisal; and *synthesis methods, such as network meta-analysis.*  **Results:** number of studies and participants identified; summary estimates with corresponding confidence/credible intervals; *treatment rankings may also be discussed. Authors may choose to summarize pairwise comparisons against a chosen treatment included in their analyses for brevity.*  **Discussion/Conclusions:** limitations; conclusions and implications of findings.  **Other:** primary source of funding; systematic review registration number with registry name. | ***Page2*** |
|  |  |  |  |
| **INTRODUCTION** |  |  |  |
| Rationale | 3 | Describe the rationale for the review in the context of what is already known*, including mention of why a network meta-analysis has been conducted.* | ***Page2-4*** |
| Objectives | 4 | Provide an explicit statement of questions being addressed, with reference to participants, interventions, comparisons, outcomes, and study design (PICOS). | ***Page4*** |
|  |  |  |  |
| **METHODS** |  |  |  |
| Protocol and registration | 5 | Indicate whether a review protocol exists and if and where it can be accessed (e.g., Web address); and, if available, provide registration information, including registration number. | ***Page4*** |
| Eligibility criteria | 6 | Specify study characteristics (e.g., PICOS, length of follow-up) and report characteristics (e.g., years considered, language, publication status) used as criteria for eligibility, giving rationale. *Clearly describe eligible treatments included in the treatment network, and note whether any have been clustered or merged into the same node (with justification).* | ***Page5*** |
| Information sources | 7 | Describe all information sources (e.g., databases with dates of coverage, contact with study authors to identify additional studies) in the search and date last searched. | ***Page4*** |
| Search | 8 | Present full electronic search strategy for at least one database, including any limits used, such that it could be repeated. | ***Page4, Supplement File2*** |
| Study selection | 9 | State the process for selecting studies (i.e., screening, eligibility, included in systematic review, and, if applicable, included in the meta-analysis). | ***Page5*** |
| Data collection process | 10 | Describe method of data extraction from reports (e.g., piloted forms, independently, in duplicate) and any processes for obtaining and confirming data from investigators. | ***Page5*** |
| Data items | 11 | List and define all variables for which data were sought (e.g., PICOS, funding sources) and any assumptions and simplifications made. | ***Page5*** |
| **Geometry of the network** | **S1** | Describe methods used to explore the geometry of the treatment network under study and potential biases related to it. This should include how the evidence base has been graphically summarized for presentation, and what characteristics were compiled and used to describe the evidence base to readers. | ***Page6*** |
| Risk of bias within individual studies | 12 | Describe methods used for assessing risk of bias of individual studies (including specification of whether this was done at the study or outcome level), and how this information is to be used in any data synthesis. | ***Page6*** |
| Summary measures | 13 | State the principal summary measures (e.g., risk ratio, difference in means). *Also describe the use of additional summary measures assessed, such as treatment rankings and surface under the cumulative ranking curve (SUCRA) values, as well as modified approaches used to present summary findings from meta-analyses.* | ***Page6*** |
| Planned methods of analysis | 14 | Describe the methods of handling data and combining results of studies for each network meta-analysis. This should include, but not be limited to:   - *Handling of multi-arm trials;* - *Selection of variance structure;* - *Selection of prior distributions in Bayesian analyses; and* - *Assessment of model fit.* | ***Page6*** |
| **Assessment of Inconsistency** | **S2** | Describe the statistical methods used to evaluate the agreement of direct and indirect evidence in the treatment network(s) studied. Describe efforts taken to address its presence when found. | ***Page6*** |
| Risk of bias across studies | 15 | Specify any assessment of risk of bias that may affect the cumulative evidence (e.g., publication bias, selective reporting within studies). | ***Page6*** |
| Additional analyses | 16 | Describe methods of additional analyses if done, indicating which were pre-specified. This may include, but not be limited to, the following:   - Sensitivity or subgroup analyses; - Meta-regression analyses; - *Alternative formulations of the treatment network; and* - *Use of alternative prior distributions for Bayesian analyses (if applicable).* | ***NA*** |
| **RESULTS†** |  |  |  |
| Study selection | 17 | Give numbers of studies screened, assessed for eligibility, and included in the review, with reasons for exclusions at each stage, ideally with a flow diagram. | ***Page7, Figure1*** |
| **Presentation of network structure** | **S3** | Provide a network graph of the included studies to enable visualization of the geometry of the treatment network. | ***Page7, Figure2*** |
| **Summary of network geometry** | **S4** | Provide a brief overview of characteristics of the treatment network. This may include commentary on the abundance of trials and randomized patients for the different interventions and pairwise comparisons in the network, gaps of evidence in the treatment network, and potential biases reflected by the network structure. | ***Page7*** |
| Study characteristics | 18 | For each study, present characteristics for which data were extracted (e.g., study size, PICOS, follow-up period) and provide the citations. | ***Page7, Table1*** |
| Risk of bias within studies | 19 | Present data on risk of bias of each study and, if available, any outcome level assessment. | ***Page7-8*** |
| Results of individual studies | 20 | For all outcomes considered (benefits or harms), present, for each study: 1) simple summary data for each intervention group, and 2) effect estimates and confidence intervals. *Modified approaches may be needed to deal with information from larger networks.* | ***Page8-9, Supplement Figure 9-10*** |
| Synthesis of results | 21 | Present results of each meta-analysis done, including confidence/credible intervals. *In larger networks, authors may focus on comparisons versus a particular comparator (e.g. placebo or standard care), with full findings presented in an appendix. League tables and forest plots may be considered to summarize pairwise comparisons.* If additional summary measures were explored (such as treatment rankings), these should also be presented. | ***Page7-8, Table2,***  ***Supplement Table1-3*** |
| **Exploration for inconsistency** | **S5** | Describe results from investigations of inconsistency. This may include such information as measures of model fit to compare consistency and inconsistency models, *P* values from statistical tests, or summary of inconsistency estimates from different parts of the treatment network. | ***Page7-8,Supplement Figure 4/7*** |
| Risk of bias across studies | 22 | Present results of any assessment of risk of bias across studies for the evidence base being studied. | ***Page7-8,Supplement Figure2/5/8*** |
| Results of additional analyses | 23 | Give results of additional analyses, if done (e.g., sensitivity or subgroup analyses, meta-regression analyses*, alternative network geometries studied, alternative choice of prior distributions for Bayesian analyses,* and so forth). | ***NA*** |
|  |  |  |  |
| **DISCUSSION** |  |  |  |
| Summary of evidence | 24 | Summarize the main findings, including the strength of evidence for each main outcome; consider their relevance to key groups (e.g., healthcare providers, users, and policy-makers). | ***Page9-11*** |
| Limitations | 25 | Discuss limitations at study and outcome level (e.g., risk of bias), and at review level (e.g., incomplete retrieval of identified research, reporting bias). *Comment on the validity of the assumptions, such as transitivity and consistency. Comment on any concerns regarding network geometry (e.g., avoidance of certain comparisons).* | ***Page11*** |
| Conclusions | 26 | Provide a general interpretation of the results in the context of other evidence, and implications for future research. | ***Page11-12*** |
|  |  |  |  |
| **FUNDING** |  |  |  |
| Funding | 27 | Describe sources of funding for the systematic review and other support (e.g., supply of data); role of funders for the systematic review. This should also include information regarding whether funding has been received from manufacturers of treatments in the network and/or whether some of the authors are content experts with professional conflicts of interest that could affect use of treatments in the network. | ***Page12,Cover letter*** |

PICOS = population, intervention, comparators, outcomes, study design.

* Text in italics indicateS wording specific to reporting of network meta-analyses that has been added to guidance from the PRISMA statement.

† Authors may wish to plan for use of appendices to present all relevant information in full detail for items in this section.

**Supplemental File 2 Search strategy**

Table 1 Search strategy in PubMed

| Step | Search strategy |
| --- | --- |
| #1 | (hip fractures[MeSH Terms]) OR (intertrochanteric fracture*[Title/Abstract]) OR (subtrochanteric fracture*[Title/Abstract]) OR (femoral neck fracture*[Title/Abstract]) OR (trochanteric fracture*[Title/Abstract]) OR (femur neck fracture*[Title/Abstract]) OR (hip fracture[Title/Abstract])) |
| #2 | ((Mind-Body Therapies[MeSH Terms]) OR (Breathing Exercises[MeSH Terms]) OR (Autogenic Training[MeSH Terms]) OR (Relaxation Therapy[MeSH Terms]) OR (Exercise[MeSH Terms]) OR (Rehabilitation[MeSH Terms]) OR (sports[MeSH Terms]) OR (Exercise Therapy[MeSH Terms]) OR (movement[MeSH Terms]) OR (early ambulation[MeSH Terms]) OR (Muscle Strength[MeSH Terms]) OR (resistance training[MeSH Terms]) OR (Circuit-Based Exercise[MeSH Terms]) OR (postural balance[MeSH Terms]) OR (occupational therapy[MeSH Terms]) OR (vibration training[Title/Abstract]) OR (Mind-Body Therapy[Title/Abstract]) OR (Mind-Body Medicine[Title/Abstract]) OR (Mind Body Medicine[Title/Abstract]) OR (Qigong[Title/Abstract]) OR (Tai Ji[Title/Abstract]) OR (Yoga[Title/Abstract]) OR (Tai Chi[Title/Abstract]) OR (Tai Ji Quan[Title/Abstract]) OR (Tai Chi Chuan[Title/Abstract]) OR (Baduanjin[Title/Abstract]) OR (Wuqinxi[Title/Abstract]) OR (Yijinjing[Title/Abstract]) OR (Physical Activit*[Title/Abstract]) OR (Physical Exercise*[Title/Abstract]) OR (Acute Exercise*[Title/Abstract]) OR (Isometric Exercise*[Title/Abstract]) OR (Aerobic Exercise*[Title/Abstract]) OR (Exercise Training*[Title/Abstract]) OR (Remedial Exercise*[Title/Abstract]) OR (Rehabilitation Exercise*[Title/Abstract]) OR (walking[Title/Abstract]) OR (training[Title/Abstract]) OR (retraining[Title/Abstract]) OR (mobili*[Title/Abstract]) OR (stepping[Title/Abstract]) OR (fall prevention exercise[Title/Abstract]) OR (foot taps[Title/Abstract]) OR (step up[Title/Abstract]) OR (gait[Title/Abstract]) OR (locomotion[Title/Abstract]) OR (motor activity[Title/Abstract]) OR (physio therap*[Title/Abstract]) OR (physical therap*[Title/Abstract]) OR (endurance[Title/Abstract]) OR (strength*[Title/Abstract]) OR (functional exercise*[Title/Abstract]) OR (ADL training[Title/Abstract]) OR (strength-promoting exercise*[Title/Abstract]) OR (stretching exercise*[Title/Abstract]) OR (standing exercise*[Title/Abstract]) OR (fexibility exercise*[Title/Abstract]) OR (Therapeutic Relaxation[Title/Abstract]) OR (Relaxation Technique[Title/Abstract]) OR (Relaxation Technic*[Title/Abstract]) OR (Nature Therap*[Title/Abstract]) OR (Ecotherap*[Title/Abstract]) OR (home rehabilitation[Title/Abstract]) OR (outpatient rehabilitation[Title/Abstract]) OR (home-based[Title/Abstract]) OR (Community exercise*[Title/Abstract]) OR (Progressive Relaxation[Title/Abstract]) OR (Strength Training*[Title/Abstract]) OR (Weight Lifting Strengthening Program*[Title/Abstract]) OR (Weight Lifting Exercise Program*[Title/Abstract]) OR (Weight-Bearing Strengthening Program*[Title/Abstract]) OR (Weight Bearing Exercise Program*[Title/Abstract]) OR (elastic tube[Title/Abstract]) OR (eccentric[Title/Abstract]) OR (concentric[Title/Abstract]) OR (pulleys[Title/Abstract]) OR (Habilitation[Title/Abstract]) OR (Circuit Based Exercise*[Title/Abstract]) OR (Circuit-Based Exercises[Title/Abstract]) OR (Circuit Training[Title/Abstract]) OR (balance exercise*[Title/Abstract]) OR (balance training[Title/Abstract]) OR (standing on one leg[Title/Abstract]) OR (balance equipment[Title/Abstract])) |
| #3 | (randomized controlled trial [pt]) OR (randomi* [tiab]) OR (randomized controlled trial [pt]) OR (controlled clinical trial [pt]) OR (randomized [tiab]) OR (randomly [tiab]) OR (trial [tiab]) OR (groups [tiab])) |
| #4 | #1 AND #2 AND #3 |

Table 2 Search strategy in Web of science

| Step | Search strategy |
| --- | --- |
| #1 | TS=(intertrochanteric fracture*) OR TS=(subtrochanteric fracture*) OR TS=(femoral neck fracture*) OR TS=(trochanteric fracture*) OR TS=(femur neck fracture*) OR TS=(hip fracture*) |
| #2 | ((TS=(vibration training) OR TS=(Mind-Body Therapy) OR TS=(Mind-Body Medicine) OR TS=(Qigong) OR TS=(Tai Ji) OR TS=(Yoga) OR TS=(Tai Chi) OR TS=(Tai Ji Quan) OR TS=(Tai Chi Chuan) OR TS=(Baduanjin) OR TS=(Wuqinxi) OR TS=(Yijinjing) OR TS=(Physical Activit*) OR TS=(Physical Exercise*) OR TS=(Acute Exercise*) OR TS=(Isometric Exercise*) OR TS=(Aerobic Exercise*) OR TS=(Exercise Training*) OR TS=(Remedial Exercise*) OR TS=(Rehabilitation Exercise*) OR TS=(walking)) OR TS=(training) OR TS=(retraining) OR TS=(mobili*) OR TS=(stepping) OR TS=(fall prevention exercise) OR TS=(foot taps) OR TS=(step up) OR TS=(gait) OR TS=(locomotion) OR TS=(motor activity) OR TS=(physio therap*) OR TS=(physical therap*) OR TS=(endurance) OR TS=(strength*) OR TS=(functional exercise*) OR TS=(ADL training) OR TS=(strength-promoting exercise*) OR TS=(stretching exercise*) OR TS=(standing exercise*) OR TS=(fexibility exercise*) OR TS=(Therapeutic Relaxation) OR TS=(Relaxation Technique) OR TS=(Relaxation Technic*) OR TS=(Nature Therap*) OR TS=(Ecotherap*) OR TS=(home rehabilitation) OR TS=(outpatient rehabilitation) OR TS=(home-based) OR TS=(Community exercise*) OR TS=(Progressive Relaxation) OR TS=(Strength Training*) OR TS=(Weight Lifting Strengthening Program*) OR TS=(Weight Lifting Exercise Program*) OR TS=(Weight-Bearing Strengthening Program*) OR TS=(Weight Bearing Exercise Program*) OR TS=(elastic tube) OR TS=(eccentric) OR TS=(concentric) OR TS=(pulleys) OR TS=(Habilitation) OR TS=(Circuit Based Exercise*) OR TS=(Circuit-Based Exercises) OR TS=(Circuit Training) OR TS=(balance exercise*) OR TS=(balance training) OR TS=(standing on one leg) OR TS=(balance equipment) OR TS=(Mind-Body Therapies) OR TS=(Breathing Exercises) OR TS=(Autogenic Training) OR TS=(Relaxation Therapy) OR TS=(Exercise) OR TS=(Rehabilitation) OR TS=(sports) OR TS=(Exercise Therapy) OR TS=(movement) OR TS=(early ambulation) OR TS=(Muscle Strength) OR TS=(resistance training) OR TS=(Circuit-Based Exercise) OR TS=(postural balance) OR TS=(occupational therapy)) |
| #3 | TS= clinical trial* OR TS=research design OR TS=comparative stud* OR TS=evaluation stud* OR TS=controlled trial* OR TS=follow-up stud* OR TS=prospective stud* OR TS=random* OR TS=placebo* OR TS=(single blind*) OR TS=(double blind*) |
| #4 | #1 AND #2 AND #3 |

Table 3 Search strategy in Embase

| Step | Search strategy |
| --- | --- |
| #1 | 'intertrochanteric fracture*':ab,ti OR 'subtrochanteric fracture*':ab,ti OR 'femoral neck fracture':ab,ti OR 'trochanteric fracture*':ab,ti OR 'fracture of hip':ab,ti OR 'femur neck fracture*':ab,ti OR 'fractures, subtrochanteric':ab,ti OR 'fractures, intertrochanteric':ab,ti OR 'fractures, trochanteric':ab,ti OR 'fractures, hip':ab,ti OR 'hip fractures':ab,ti OR 'femoral neck fractures':ab,ti |
| #2 | 'vibration training':ab,ti OR 'mind-body therapy':ab,ti OR 'mind-body medicine':ab,ti OR 'mind body medicine':ab,ti OR 'qigong':ab,ti OR 'tai ji':ab,ti OR 'yoga':ab,ti OR 'tai chi':ab,ti OR 'tai ji quan':ab,ti OR 'tai chi chuan':ab,ti OR 'baduanjin':ab,ti OR 'wuqinxi':ab,ti OR 'yijinjing':ab,ti OR 'physical activit*':ab,ti OR 'physical exercise*':ab,ti OR 'acute exercise*':ab,ti OR 'isometric exercise*':ab,ti OR 'aerobic exercise*':ab,ti OR 'exercise training*':ab,ti OR 'remedial exercise*':ab,ti OR 'rehabilitation exercise*':ab,ti OR 'walking':ab,ti OR 'retraining or mobili*':ab,ti OR 'stepping':ab,ti OR 'fall prevention exercise':ab,ti OR 'foot taps':ab,ti OR 'step up':ab,ti OR 'gait':ab,ti OR 'locomotion':ab,ti OR 'motor activity':ab,ti OR 'physio therap*':ab,ti OR 'physical therap*':ab,ti OR 'endurance':ab,ti OR 'strength*':ab,ti OR 'functional exercise*':ab,ti OR 'ADL training':ab,ti OR 'strength-promoting exercise*':ab,ti OR 'stretching exercise*':ab,ti OR 'standing exercise*':ab,ti OR 'fexibility exercise*':ab,ti OR 'therapeutic relaxation':ab,ti OR 'relaxation technique':ab,ti OR 'relaxation technic*':ab,ti OR 'nature therap*':ab,ti OR 'ecotherap*':ab,ti OR 'home rehabilitation':ab,ti OR 'outpatient rehabilitation':ab,ti OR 'home-based':ab,ti OR 'community exercise*':ab,ti OR 'progressive relaxation':ab,ti OR 'strength training*':ab,ti  OR 'weight lifting strengthening program*':ab,ti OR 'weight lifting exercise program*':ab,ti OR 'weight-bearing strengthening program*':ab,ti OR 'weight bearing exercise program*':ab,ti OR 'elastic tube':ab,ti OR 'eccentric':ab,ti OR 'concentric':ab,ti OR 'pulleys':ab,ti OR 'habilitation':ab,ti OR 'circuit based exercise*':ab,ti OR 'circuit-based exercises':ab,ti OR 'circuit training':ab,ti OR 'balance exercise*':ab,ti OR 'balance training':ab,ti OR 'standing on one leg':ab,ti OR 'balance equipment':ab,ti OR 'mind-body therapies':ab,ti OR 'breathing exercises':ab,ti OR 'autogenic training':ab,ti OR 'relaxation therapy':ab,ti OR 'exercise therapy':ab,ti OR 'movement':ab,ti OR 'early ambulation':ab,ti OR  'muscle strength':ab,ti OR 'resistance training':ab,ti OR 'circuit-based exercise':ab,ti OR 'postural balance':ab,ti OR 'occupational therapy':ab,ti |
| #3 | ('crossover procedure':de OR 'double-blind procedure':de OR 'randomized controlled trial':de) AND or  AND 'single-blind procedure':de OR (random*:de,ab,ti AND or :de,ab,ti AND factorial*:de,ab,ti) OR crossover*:de,ab,ti OR ((cross NEXT/1 over*):de,ab,ti) OR placebo*:de,ab,ti OR ((doubl* NEAR/1 blind*):de,ab,ti) OR ((singl* NEAR/1 blind*):de,ab,ti) OR assign*:de,ab,ti OR allocat*:de,ab,ti OR volunteer*:de,ab,ti |
| #4 | 'hip fracture'/exp |
| #5 | #1 AND #4 |
| #6 | 'training'/exp |
| #7 | 'sport'/exp |
| #8 | 'exercise'/exp |
| #9 | 'rehabilitation'/exp |
| #10 | #2 OR #6 OR #7 OR #8 OR #9 |
| #11 | #3 AND #5 AND #10 |

Table 4 Search strategy in Cochrane

| Step | Search strategy |
| --- | --- |
| #1 | MeSH descriptor: [Hip Fractures] explode all trees |
| #2 | (intertrochanteric fracture*):ti,ab,kw OR (subtrochanteric fracture*):ti,ab,kw OR (femoral neck fracture*):ti,ab,kw OR (trochanteric fracture*):ti,ab,kw OR (femur neck fracture*):ti,ab,kw (Word variations have been searched) |
| #3 | #1 OR #2 |
| #4 | MeSH descriptor: [Exercise] explode all trees |
| #5 | MeSH descriptor: [Sports] explode all trees |
| #6 | MeSH descriptor: [Teaching] explode all trees |
| #7 | MeSH descriptor: [Rehabilitation] explode all trees |
| #8 | (vibration training):ti,ab,kw OR (Mind-Body Therapy):ti,ab,kw OR (Mind-Body Medicine):ti,ab,kw OR (Qigong):ti,ab,kw OR (Tai Ji):ti,ab,kw (Word variations have been searched) |
| #9 | (Yoga):ti,ab,kw OR (Tai Chi):ti,ab,kw OR (Tai Ji Quan):ti,ab,kw OR (Tai Chi Chuan):ti,ab,kw OR (Baduanjin):ti,ab,kw (Word variations have been searched) |
| #10 | (Wuqinxi):ti,ab,kw OR (Yijinjing):ti,ab,kw OR (Physical Activit*):ti,ab,kw OR (Physical Exercise*):ti,ab,kw OR (Acute Exercise*):ti,ab,kw (Word variations have been searched) |
| #11 | (Isometric Exercise*):ti,ab,kw OR (Aerobic Exercise*):ti,ab,kw OR (Exercise Training*):ti,ab,kw OR (Remedial Exercise*):ti,ab,kw OR (Rehabilitation Exercise*):ti,ab,kw (Word variations have been searched) |
| #12 | (walking):ti,ab,kw OR (mobili*):ti,ab,kw OR (stepping):ti,ab,kw OR (fall prevention exercise):ti,ab,kw OR (gait):ti,ab,kw (Word variations have been searched) |
| #13 | (locomotion):ti,ab,kw OR (motor activity):ti,ab,kw OR (physio therap*):ti,ab,kw OR (physical therap*):ti,ab,kw OR (endurance):ti,ab,kw (Word variations have been searched) |
| #14 | (strength*):ti,ab,kw OR (functional exercise*):ti,ab,kw OR (ADL training):ti,ab,kw OR (strength-promoting exercise*):ti,ab,kw OR (stretching exercise*):ti,ab,kw (Word variations have been searched) |
| #15 | (standing exercise*):ti,ab,kw OR (fexibility exercise*):ti,ab,kw OR (Therapeutic Relaxation):ti,ab,kw OR (home rehabilitation):ti,ab,kw OR (outpatient rehabilitation):ti,ab,kw (Word variations have been searched) |
| #16 | (home-based):ti,ab,kw OR (Community exercise*):ti,ab,kw OR (Progressive Relaxation):ti,ab,kw OR (Strength Training*):ti,ab,kw OR (Weight Bearing):ti,ab,kw (Word variations have been searched) |
| #17 | (Circuit Based Exercise*):ti,ab,kw OR (Circuit Training):ti,ab,kw OR (balance exercise*):ti,ab,kw OR (balance training):ti,ab,kw OR (Mind-Body Therapies):ti,ab,kw (Word variations have been searched) |
| #18 | (Breathing Exercises):ti,ab,kw OR (Autogenic Training):ti,ab,kw OR (Relaxation Therapy):ti,ab,kw OR (Exercise Therapy):ti,ab,kw OR (movement):ti,ab,kw (Word variations have been searched) |
| #19 | (early ambulation):ti,ab,kw OR (Muscle Strength):ti,ab,kw OR (resistance training):ti,ab,kw OR (Circuit-Based Exercise):ti,ab,kw OR (postural balance):ti,ab,kw (Word variations have been searched) |
| #20 | (occupational therapy):ti,ab,kw (Word variations have been searched) |
| #21 | #4 OR #5 OR #6 OR #7 OR #8 OR #9 OR #10 OR #11 OR #12 OR #13 OR #14 OR #15 OR #16 OR #17 OR #18 OR #19 OR #20 |
| #22 | #3 AND #21 |

Table 5 Search strategy in CINAHL

| Step | Search strategy |
| --- | --- |
| #1 | (hip fractures) OR (intertrochanteric fracture*) OR (subtrochanteric fracture*) OR (femoral neck fracture*) OR (trochanteric fracture*) OR (femur neck fracture*) OR (hip fracture)) |
| #2 | ((Mind-Body Therapies) OR (Breathing Exercises) OR (Autogenic Training) OR (Relaxation Therapy) OR (Exercise) OR (Rehabilitation) OR (sports) OR (Exercise Therapy) OR (movement) OR (early ambulation) OR (Muscle Strength) OR (resistance training) OR (Circuit-Based Exercise) OR (postural balance) OR (occupational therapy) OR (vibration training) OR (Mind-Body Therapy) OR (Mind-Body Medicine) OR (Mind Body Medicine) OR (Qigong) OR (Tai Ji) OR (Yoga) OR (Tai Chi) OR (Tai Ji Quan) OR (Tai Chi Chuan) OR (Baduanjin) OR (Wuqinxi) OR (Yijinjing) OR (Physical Activit*) OR (Physical Exercise*) OR (Acute Exercise*) OR (Isometric Exercise*) OR (Aerobic Exercise*) OR (Exercise Training*) OR (Remedial Exercise*) OR (Rehabilitation Exercise*) OR (walking) OR (training) OR (retraining) OR (mobili*) OR (stepping) OR (fall prevention exercise) OR (foot taps) OR (step up) OR (gait) OR (locomotion) OR (motor activity) OR (physio therap*) OR (physical therap*) OR (endurance) OR (strength*) OR (functional exercise*) OR (ADL training) OR (strength-promoting exercise*) OR (stretching exercise*) OR (standing exercise*) OR (fexibility exercise*) OR (Therapeutic Relaxation) OR (Relaxation Technique) OR (Relaxation Technic*) OR (Nature Therap*) OR (Ecotherap*) OR (home rehabilitation) OR (outpatient rehabilitation) OR (home-based) OR (Community exercise*) OR (Progressive Relaxation) OR (Strength Training*) OR (Weight Lifting Strengthening Program*) OR (Weight Lifting Exercise Program*) OR (Weight-Bearing Strengthening Program*) OR (Weight Bearing Exercise Program*) OR (elastic tube) OR (eccentric) OR (concentric) OR (pulleys) OR (Habilitation) OR (Circuit Based Exercise*) OR (Circuit-Based Exercises) OR (Circuit Training) OR (balance exercise*) OR (balance training) OR (standing on one leg) OR (balance equipment)) |
| #3 | (randomized controlled trial) OR (randomi*) OR (randomized controlled trial) OR (controlled clinical trial) OR (randomized) OR (randomly) OR (trial) OR (groups)) |
| #4 | #1 AND #2 AND #3 |

Table 6 Search strategy in CNKI

| Step | Search strategy |
| --- | --- |
| #1 | SU=(hip fracture+hip joint fracture+fracture of femoral neck+intertrochanteric fracture+femoral subtrochanteric fracture+hip fragility fracture+osteoporosis fracture) |
| #2 | SU=(motion+movement+locomotion+sport+kinesis+exercise+training+physical training+physical function+rehabilitation+resistance+balance+oscillation+vibration+  relaxation+weight-bearing+tolerance+endurance+capacity+aerobic+tai chi  +yoga+qigong+baduanjin+eight-sectioned exercise+eight-section brocade+  five mimic-animal+changing tendon+yijinjing+walking+recovery of joint+muscle strength+physical therapy+athletic sport+activity+strength training+power training+physical strength+task-oriented training+body exercise+functional state+occupational therapy) |
| #3 | SU=(randomized+clinical experiment+clinical trial+application+utilization+influence  +impact+affect+result+effect+curative importance+curative effectiveness+curative efficacy+intervention+observation+analysis+RCT+CCT) |
| #4 | #1 AND #2 AND #3 |

Table 7 Search strategy in WanFang

| Step | Search strategy |
| --- | --- |
| #1 | Title or Keywords:"hip fracture" or "hip joint fracture" or "fracture of femoral neck" or "intertrochanteric fracture" or "femoral subtrochanteric fracture" or "hip fragility fracture" or "osteoporosis fracture" |
| #2 | Title or Keywords:"motion" or "movement" or "locomotion" or "sport" or "kinesis" or "exercise" or "training" or "physical training" or "physical function" or "rehabilitation" or "resistance" or "balance" or "oscillation" or "vibration or relaxation" or "weight-bearing" or "tolerance" or "endurance" or "capacity" or "aerobic" or "tai chi" or "yoga" or "qigong" or "baduanjin" or "eight-sectioned exercise" or "eight-section brocade" or "five mimic-animal" or "changing tendon" or "yijinjing" or "walking" or "recovery of joint" or "muscle strength" or "physical therapy" or "athletic sport" or "activity" or "strength training" or "power training" or "physical strength" or "task-oriented training" or "body exercise" or "functional state" or "occupational therapy" |
| #3 | Title or Keywords:"randomized" or "clinical experiment" or "clinical trial" or "application" or "utilization" or "influence" or "impact" or "affect" or "result" or "effect" or "curative importance" or "curative effectiveness" or "curative efficacy" or "intervention" or "observation" or "analysis" or "RCT" or "CCT" |
| #4 | #1 AND #2 AND #3 |

Table 8 Search strategy in VIP

| Step | Search strategy |
| --- | --- |
| #1 | M=("hip fracture" or "hip joint fracture" or "fracture of femoral neck" or "intertrochanteric fracture" or "femoral subtrochanteric fracture" or "hip fragility fracture" or "osteoporosis fracture") |
| #2 | M=("motion" or "movement" or "locomotion" or "sport" or "kinesis" or "exercise" or "training" or "physical training" or "physical function" or "rehabilitation" or "resistance" or "balance" or "oscillation" or "vibration or relaxation" or "weight-bearing" or "tolerance" or "endurance" or "capacity" or "aerobic" or "tai chi" or "yoga" or "qigong" or "baduanjin" or "eight-sectioned exercise" or "eight-section brocade" or "five mimic-animal" or "changing tendon" or "yijinjing" or "walking" or "recovery of joint" or "muscle strength" or "physical therapy" or "athletic sport" or "activity" or "strength training" or "power training" or "physical strength" or "task-oriented training" or "body exercise" or "functional state" or "occupational therapy") |
| #3 | M=("randomized" or "clinical experiment" or "clinical trial" or "application" or "utilization" or "influence" or "impact" or "affect" or "result" or "effect" or "curative importance" or "curative effectiveness" or "curative efficacy" or "intervention" or "observation" or "analysis" or "RCT" or "CCT") |
| #4 | #1 AND #2 AND #3 |

Table 9 Search strategy in CBM

| Step | Search strategy |
| --- | --- |
| #1 | Theme:(hip fracture OR hip joint fracture OR fracture of femoral neck OR intertrochanteric fracture OR femoral subtrochanteric fracture OR hip fragility fracture OR osteoporosis fracture) |
| #2 | Theme:(motion OR movement OR locomotion OR sport OR kinesis OR exercise OR training OR physical training OR physical function OR rehabilitation OR resistance OR balance OR oscillation OR vibration OR relaxation OR weight-bearing OR tolerance OR endurance OR capacity OR aerobic OR tai chi OR yoga OR qigong OR baduanjin OR eight-sectioned exercise OR eight-section brocade OR five mimic-animal OR changing tendon OR yijinjing OR walking OR recovery of joint OR muscle strength OR physical therapy OR athletic sport OR activity OR strength training OR power training OR physical strength OR task-oriented training OR body exercise OR functional state OR occupational therapy) |
| #3 | Theme:(randomized OR clinical experiment OR clinical trial OR application OR utilization OR influence OR impact OR affect OR result OR effect OR curative importance OR curative effectiveness OR curative efficacy OR intervention OR observation OR analysis OR RCT OR CCT) |
| #4 | #1 AND #2 AND #3 |

**Supplemental File 3**

**Reference list of included studies**

1. Sherrington C, Lord SR. Home exercise to improve strength and walking velocity after hip fracture: a randomized controlled trial. *Arch Phys Med Rehabil*. 1997;**78**(2):208-212. doi:10.1016/s0003-9993(97)90265-3
2. Hauer K, Specht N, Schuler M, Bärtsch P, Oster P. Intensive physical training in geriatric patients after severe falls and hip surgery. *Age Ageing*. 2002;**31**(1):49-57. doi:10.1093/ageing/31.1.49
3. Sherrington C, Lord SR, Herbert RD. A randomised trial of weight-bearing versus non-weight-bearing exercise for improving physical ability in inpatients after hip fracture. *Aust J Physiother*. 2003;**49**(1):15-22. doi:10.1016/s0004-9514(14)60184-7
4. Binder EF, Brown M, Sinacore DR, Steger-May K, Yarasheski KE, Schechtman KB. Effects of extended outpatient rehabilitation after hip fracture: a randomized controlled trial. *JAMA*. 2004;**292**(7):837-846. doi:10.1001/jama.292.7.837
5. Sherrington C, Lord SR, Herbert RD. A randomized controlled trial of weight-bearing versus non-weight-bearing exercise for improving physical ability after usual care for hip fracture. *Arch Phys Med Rehabil*. 2004;**85**(5):710-716. doi:10.1016/s0003-9993(03)00620-8
6. Suetta C, Magnusson SP, Rosted A, et al. Resistance training in the early postoperative phase reduces hospitalization and leads to muscle hypertrophy in elderly hip surgery patients--a controlled, randomized study. *J Am Geriatr Soc*. 2004;**52**(12):2016-2022. doi:10.1111/j.1532-5415.2004.52557.x
7. Mangione KK, Craik RL, Tomlinson SS, Palombaro KM. Can elderly patients who have had a hip fracture perform moderate- to high-intensity exercise at home?. *Phys Ther*. 2005;**85**(8):727-739. PMID: 16048421
8. Mendelsohn ME, Overend TJ, Connelly DM, Petrella RJ. Improvement in aerobic fitness during rehabilitation after hip fracture. *Arch Phys Med Rehabil*. 2008;**89**(4):609-617. doi:10.1016/j.apmr.2007.09.036
9. Minna Mard, Johanna Vaha, Heinonen A. The effects of muscle strength and power training on mobility among older hip fracture patients. *Advances in Physiotherapy.* 2008;**10**(4): 195-202. doi: 10.1080/14038190801999570
10. Portegijs E, Kallinen M, Rantanen T, et al. Effects of resistance training on lower-extremity impairments in older people with hip fracture. *Arch Phys Med Rehabil*. 2008;**89**(9):1667-1674. doi:10.1016/j.apmr.2008.01.026
11. Mangione KK, Craik RL, Palombaro KM, Tomlinson SS, Hofmann MT. Home-based leg-strengthening exercise improves function 1 year after hip fracture: a randomized controlled study. *J Am Geriatr Soc*. 2010;**58**(10):1911-1917. doi:10.1111/j.1532-5415.2010.03076.x

12. Kui DQ. The effect of early weight-bearing and walking on elderly patients after internal fixation of femoral neck fractures. *Chin J Phys Med Rehabil.* 2011;(08): 614-616. doi:10.3760/cma.j.issn.0254-1424.2011.08.015

13. Yan Y. Balanced functional exercise in the rehabilitation of elderly patients with femoral neck fractures. *Chin J Mod Nur.* 2012;(24): 2938-2939.

14. Singh N A, Quine S, Clemson L M, et al. Effects of high-intensity progressive resistance training and targeted multidisciplinary treatment of frailty on mortality and nursing home admissions after hip fracture: a randomized controlled trial. *J Am Med Dir Assoc.* 2012;**13**(1): 24-30. doi:10.1016/j.jamda.2011.08.005

15. Sylliaas H, Brovold T, Wyller T B, et al. Prolonged strength training in older patients after hip fracture: a randomized controlled trial. *Age Ageing*. 2012;**41**(2): 206-212. doi:10.1093/ageing/afr164

16. Morishima Y, Mizushima T, Yamauchi K, et al. Effects of home-based interval walking training on thigh muscle strength and aerobic capacity in female total hip arthroplasty patients: a randomized, controlled pilot study. *Plos One.* 2014; **9**(9): e108690. doi:10.1371/journal.pone.0108690

17. Xing S, Zhang LL, Tang SY, et al. The design and application of household music rehabilitation exercises in the post-operation for elderly patient with hip fracture. *Orthop J Chin*. 2016;**24**(05): 470-473.

18. Zhao X. Application of home furnishing music rehabilitation gymnastics in discharged elderly patients with hip fracture. *Chin Nurs Res.* 2016;**30**(24): 3056-3058. doi:10.3969/j.issn.1009-6493.2016.24.039

19. van Ooijen MW, Roerdink M, Trekop M, Janssen TW, Beek PJ. The efficacy of treadmill training with and without projected visual context for improving walking ability and reducing fall incidence and fear of falling in older adults with fall-related hip fracture: a randomized controlled trial. *BMC Geriatr*. 2016;**16**(1):215. doi:10.1186/s12877-016-0388-x

20. Xu YF. Rehabilitation effects of the comprehensive intervention of home-based balance training on the elderly with femoral neck fracture. *Chin J Mod Nurs.* 2017;**23**(13): 1797-1800. doi:10.3760/cma.j.issn.1674-2907.2017.13.022

21. Zhang T, Xie Q, Wang J, et al. Effect of Intensive Core Muscles Training on General Function in Patients after Hip Arthroplasty. *Chin J Rehabil Theor and Prac.* 2017;**23**(10): 1171-1175. doi:10.3969/j.issn.1006-9771.2017.10.010

22. Monticone M, Ambrosini E, Brunati R, et al. How balance task-specific training contributes to improving physical function in older subjects undergoing rehabilitation following hip fracture: a randomized controlled trial. *Clin Rehabil.* 2018;**32**(3): 340-351. doi:10.1177/0269215517724851

23. Dong GM, Zhang W. Effect of Relaxation Training combined with Muscle Group Intensive Training on Patients with Hip Replacement.  *Qilu J Nurs.* 2019;25(20): 19-22. doi:10.3969/j.issn.1006-7256.2019.20.006

24. Kang ZM, Xi GQ, Chen YF, et al. Effect of hip rehabilitation exercise on functional rehabilitation of hip fracture patients after operation. *Chin J Prim Med Pharm.* 2019;**26**(16): 2041-2044. doi:10.3760/cma.j.issn.1008-6706.2019.16.032

25. Wang G, Liu HJ, Li ZW, et al. Role of progressive resistance exercise in the rehabilitation of femoral intertrochanteric fracture after operation. *Journal of Zhengzhou University (Medical Sciences).* 2019;**54**(04): 615-618.

26. Wu WY, Xu WG. Evaluation of the effect of early total weight-bearing training after cementless total hip arthroplasty. *Chin J Tiss Engin Res.* 2019;**23**(18): 2827-2832. doi:10.3969/j.issn.2095-4344.1196

27. Stasi S, Papathanasiou G, Chronopoulos E, et al. The Effect of Intensive Abductor Strengthening on Postoperative Muscle Efficiency and Functional Ability of Hip-Fractured Patients: A Randomized Controlled Trial. *Indian J Orthop.* 2019;**53**(3): 407-419. doi:10.4103/ortho.IJOrtho_183_18

28. Cai Y, Wang J, Cen WX, et al. Effect of progressive resistance training on rehabilitation of patients with osteoporotic femoral neck fracture after hip arthroplasty. *Chin J Frontiers Med Sci*. 2020;**12**(11): 68-71.

29. Chi K, Liu B, Yang XM. Application of early total weight bearing training in biological total hip replacement for elderly patients with unstable intertrochanteric fracture of femur. *Chin J Frontiers Med Sci.* 2020;**12**(12): 42-45.

30. Li JH. Effect of intensive training of core muscle group on overall functional recovery in patients undergoing hip arthroplasty. *IMHGN.* 2020; **26**(12): 1765-1767. doi:10.3760/cma.j.issn.1007-1245.2020.12.035

31. Qin DJ, Zhang Y, Zhang HF. Application of home furnishing music rehabilitation gymnastics in elderly patients discharged from hospital after intertrochanteric fracture of the femur. *Henan Med Res*. 2020;**29**(35): 6689-6691. doi:10.3969/j.issn.1004-437X.2020.35.067

32. Sun ZL, Xu R. The effect of core muscle group training on rehabilitation and complications of elderly patients with femoral neck fracture after joint replacement. *Anhui J Med and Pharm.* 2020;**24**(07): 1359-1362. doi:10.3969/j.issn.1009-6469.2020.07.022

33. Wang K, Zhang Z, Du YK. Effect of early weight-bearing and walking on patients with femoral neck fracture after internal fixation. *Contemp Med.* 2020;**26**(35): 50-53. doi:10.3969/j.issn.1009-4393.2020.35.020

34. Xu LJ. Effect of home-based balance exercises on postoperative hip function, balance and compliance in elderly patients with femoral neck fractures. *Henan Med Res*. 2020;**29**(33): 6323-6324. doi:10.3969/j.issn.1004-437X.2020.33.074

35. Kim P, Lee H, Choi W, et al. Effect of 4 Weeks of Anti-Gravity Treadmill Training on Isokinetic Muscle Strength and Muscle Activity in Adults Patients with a Femoral Fracture: A Randomized Controlled Trial. *Int J Environ Res Public Health.* 2020;**17**(22). doi:10.3390/ijerph17228572

36. Oh M K, Yoo J I, Byun H, et al. Efficacy of Combined Antigravity Treadmill and Conventional Rehabilitation After Hip Fracture in Patients with Sarcopenia. *J Gerontol A Biol Sci Med Sci*. 2020; 75(10): 173-181. doi:10.1093/gerona/glaa158

37. Ding XY, Li J. Application of " Four More" family rehabilitation exercise in elderly patients with hip fracture. *Int J Nurs.* 2021;**40**(11):1973-1975. doi:10.3760/cma.j.cn221370-20200330-00571

38. Guo YT, Wang JW. Application of early rehabilitation exercise combined with family balance training in postoperative rehabilitation for elderly patients with femoral neck fracture. *Chin J Convale Med*. 2021;**30**(03): 266-268.

39. Liu Q, Li SS, Li XZ, et al. Effect of home-based balance training on hip function, lower limb mobility and balance in elderly patients with Pauwels type III femoral neck fractures. *Chin Gen Pract Nurs.* 2021;**19**(36):5110-5113. doi:10.12104/j.issn.1674-4748.2021.36.017

40. Wang DX, Lin Y, Zhang T, et al. Application of self-made rehabilitation exercises in postoperative rehabilitation of elderly patients with hip fracture. *Chin Evid Based Nurs.* 2021; **7**(15):2059-2061. doi:10.12102/j.issn.2095-8668.2021.15.015

41. Xu XX, Yang LF, Jing S, et al. Application effect of progressive resistance exercise training in elderly patients with intertrochanteric fracture after internal fixation. *Chin Med Herald*. 2021;**18**(09): 99-103.

42. Corna S, Arcolin I, Giardini M, et al. Addition of aerobic training to conventional rehabilitation after hip fracture: a randomized, controlled, pilot feasibility study. *Clin Rehabil,* 2021;**35**(4): 568-577. doi:10.1177/0269215520968694

43. Paulsson M, Ekholm C, Jonsson E, et al. Immediate Full Weight-Bearing Versus Partial Weight-Bearing After Plate Fixation of Distal Femur Fractures in Elderly Patients. A Randomized Controlled Trial. *Geriatr Orthop Surg Rehabil.* 2021;**12**: 21514593211055889. doi:10.1177/21514593211055889

44. Li J, Li J, Zhang QL. Effect of core muscle strengthening training on functional recovery and Barthel Index in hip replacement patients. *Jilin J Med.* 2022; **43**(03): 785-786. doi:10.3969/j.issn.1004-0412.2022.03.084

45. Yan YY, Zhang Y. Effect of progressive resistance exercise training on hip function after internal fixation of femoral intertrochanteric fractures in elderly patients. *Clin Res Prac*. 2022;**7**(20): 169-171.

46. Overgaard J A, Kallemose T, Mangione K K, et al. Six Versus 12 Weeks of Outpatient Physical Therapy Including Progressive Resistance Training in Cognitively Intact Older Adults After Hip Fracture: A Multicenter Randomized Controlled Trial. *J Gerontol A Biol Sci Med Sci*. 2022;**77**(7): 1455-1462. doi:10.1093/gerona/glab256


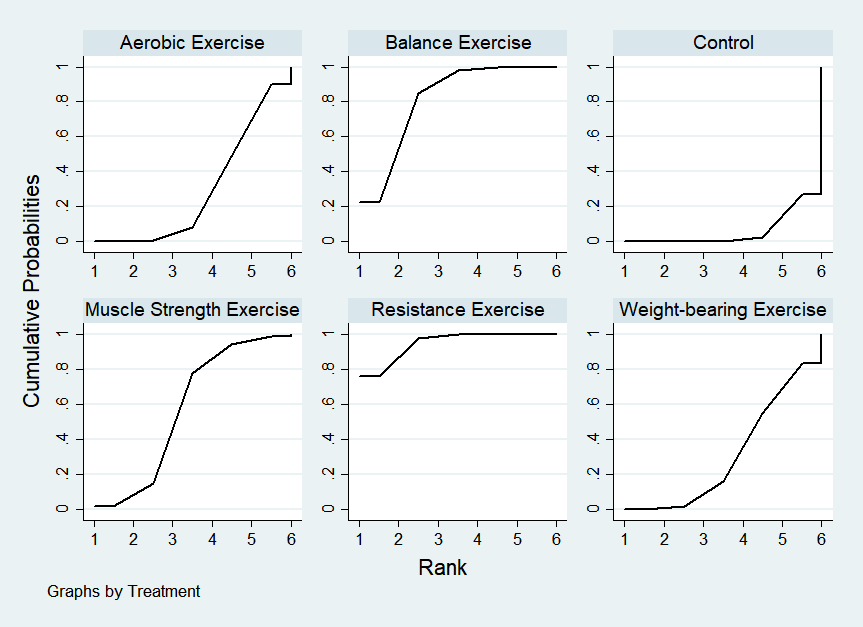


**Supplement Figure 1** Plots of the surface under the cumulative ranking curves (SUCRA) for all comparisons in the hip function

**
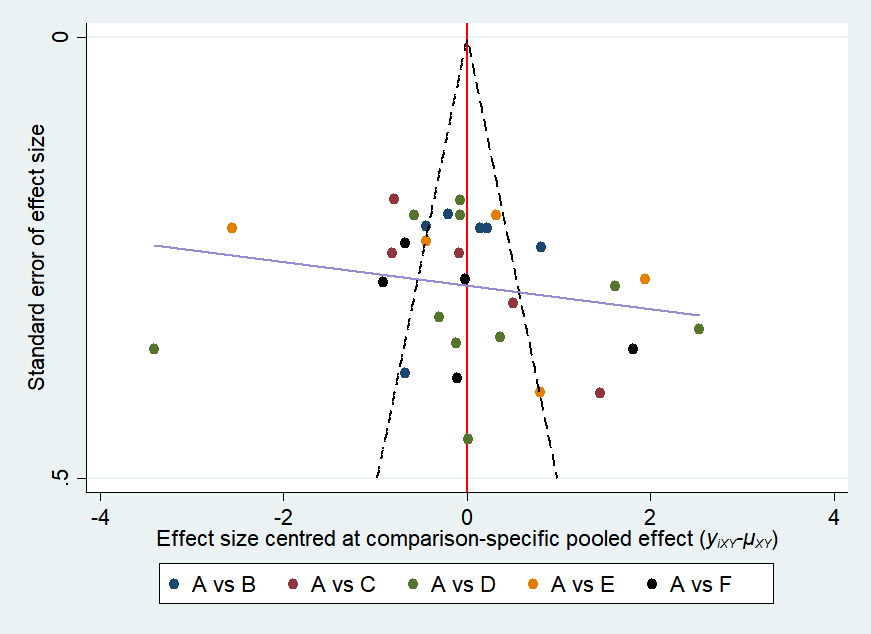
**

**Supplement Figure 2** Funnel plots for the effects of exercise interventions on hip function for elderly patients with hip fracture. A, control group; B, resistance exercise; C, balance exercise; D, aerobic exercise; E, weight-bearing exercise; F, muscle strength exercise.


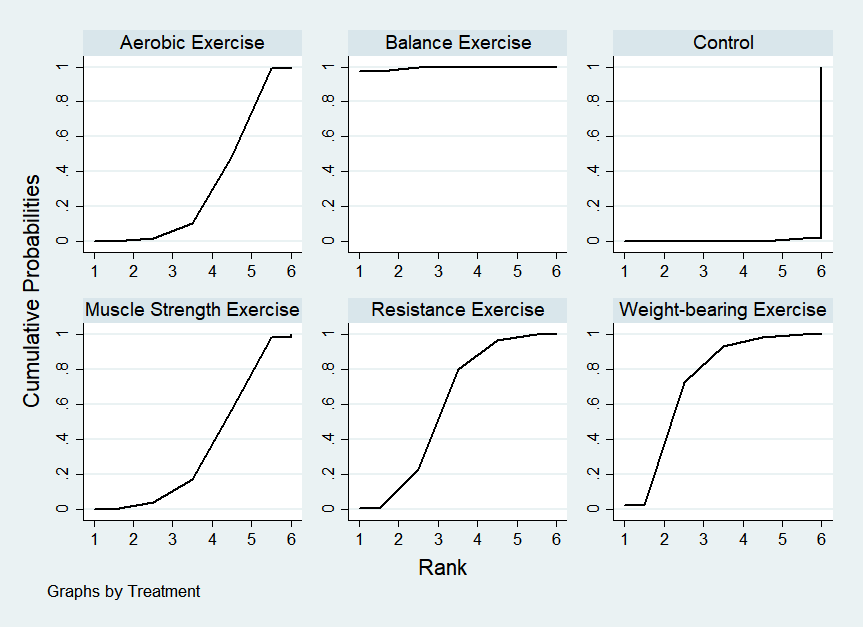


**Supplement Figure 3** Plots of the surface under the cumulative ranking curves (SUCRA) for all comparisons in the ADL


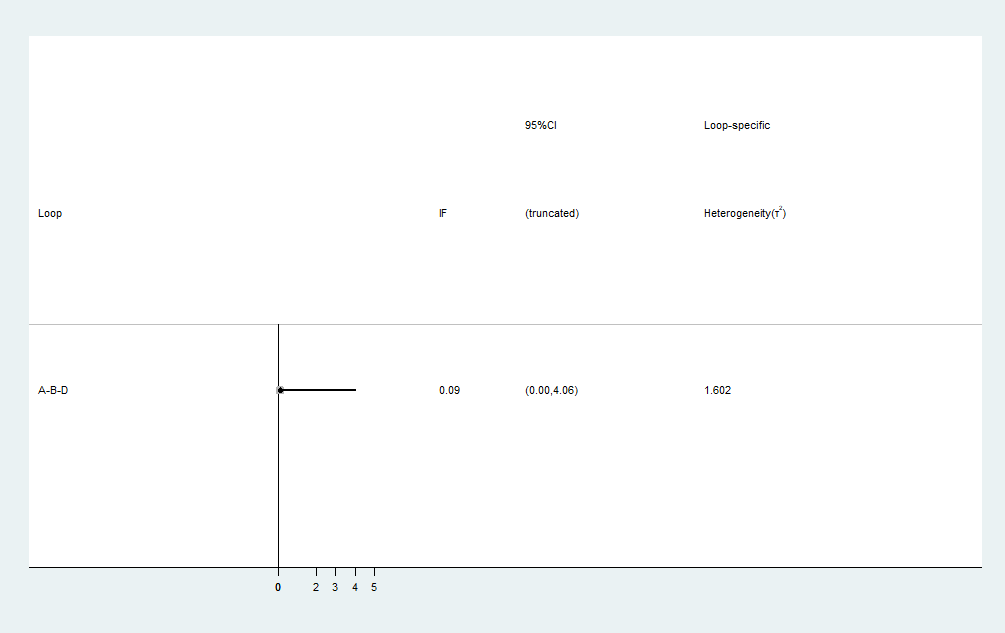


**Supplement Figure 4** Inconsistency plot for the ADL network, assuming loop-specific

heterogeneity estimates. A, control group; B, resistance exercise; D, balance exercise.


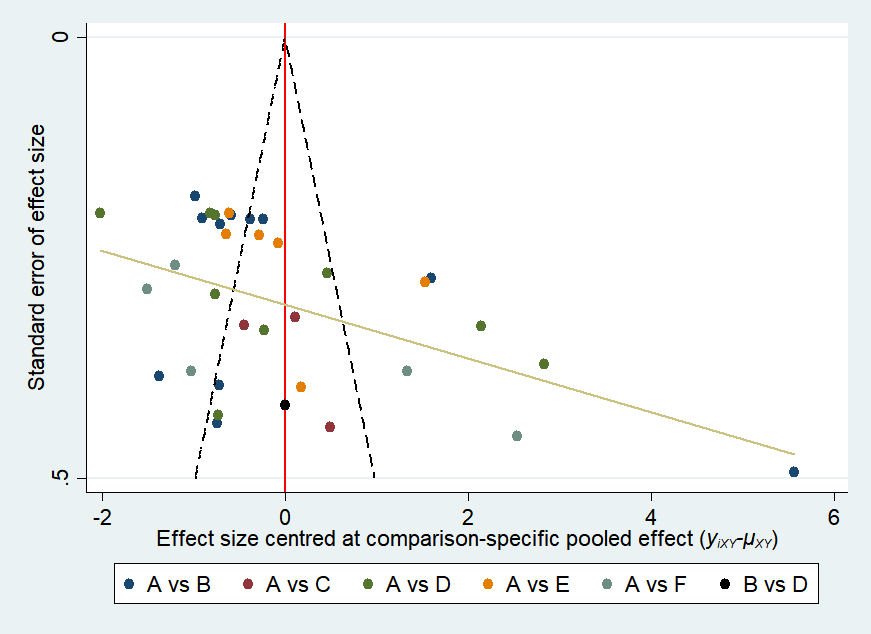


**Supplement Figure 5** Funnel plots for the effects of exercise interventions on ADL for elderly patients with hip fracture. A, control group; B, resistance exercise; C, balance exercise; D, aerobic exercise; E, weight-bearing exercise; F, muscle strength exercise.


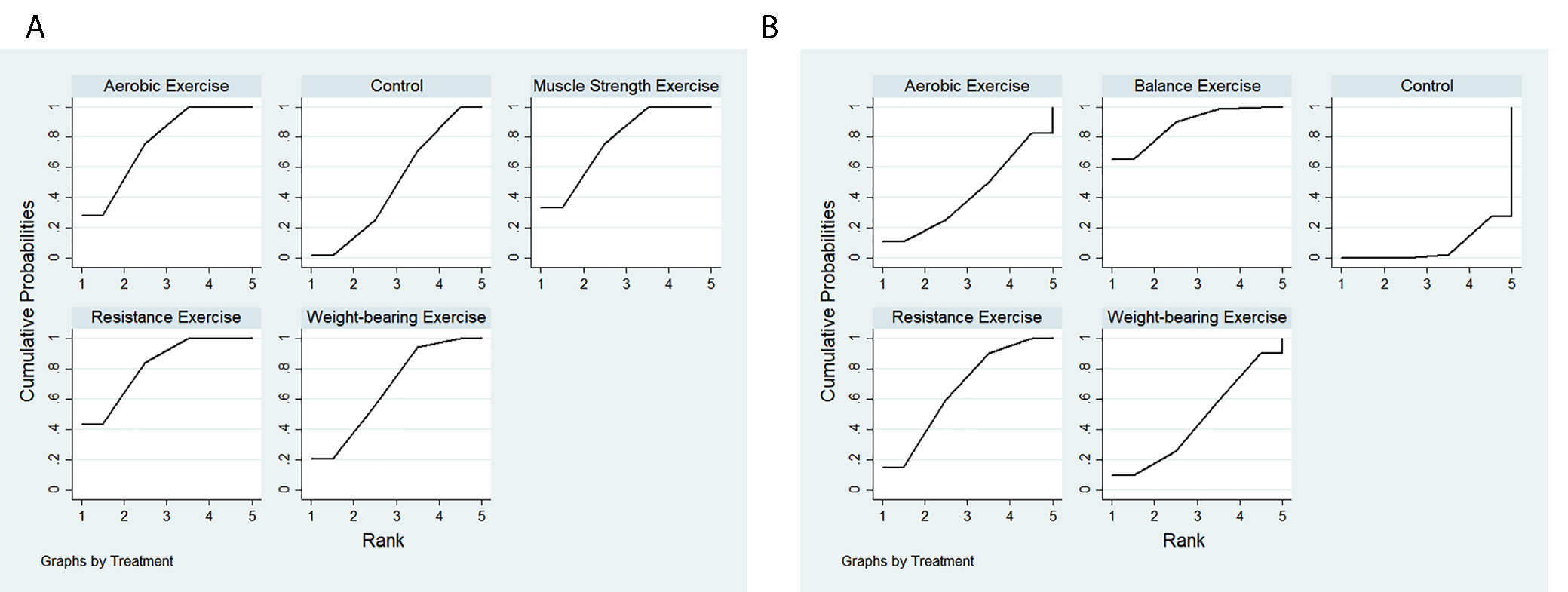


**Supplement Figure 6** Plots of the surface under the cumulative ranking curves (SUCRA) for all comparisons in walking capacity (A) and balance ability (B).


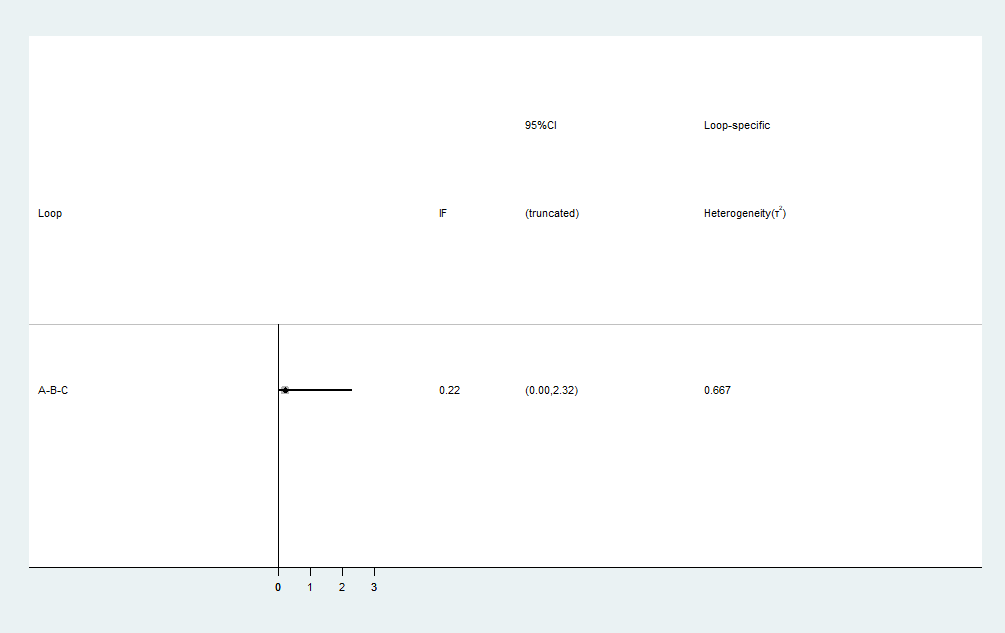


**Supplement Figure 7** Inconsistency plot for the walking capacity network, assuming loop-specific heterogeneity estimates. A, control group; B, resistance exercise; C, aerobic exercise.


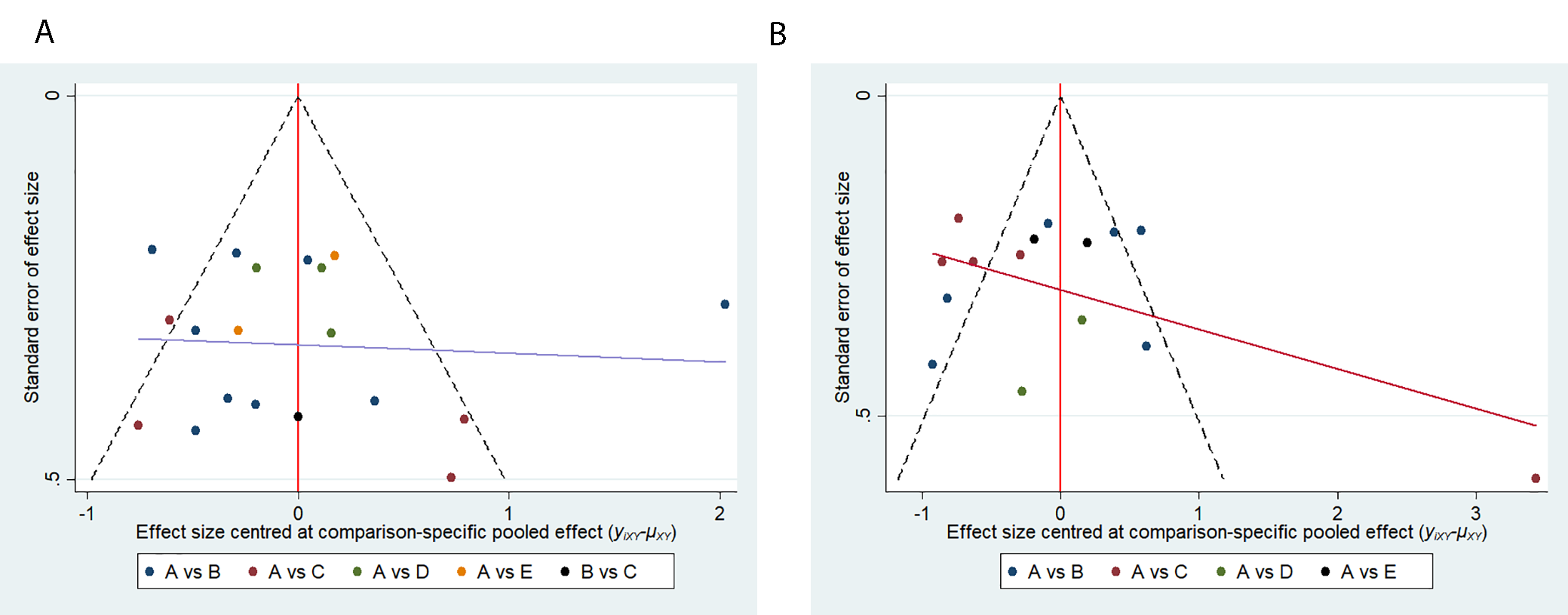


**Supplement Figure 8** Funnel plots for the effects of exercise interventions on walking capacity (A) and balance ability (B) for elderly patients with hip fracture. A, control group; B, resistance exercise; C, aerobic exercise; D, weight-bearing exercise; E, muscle strength exercise.


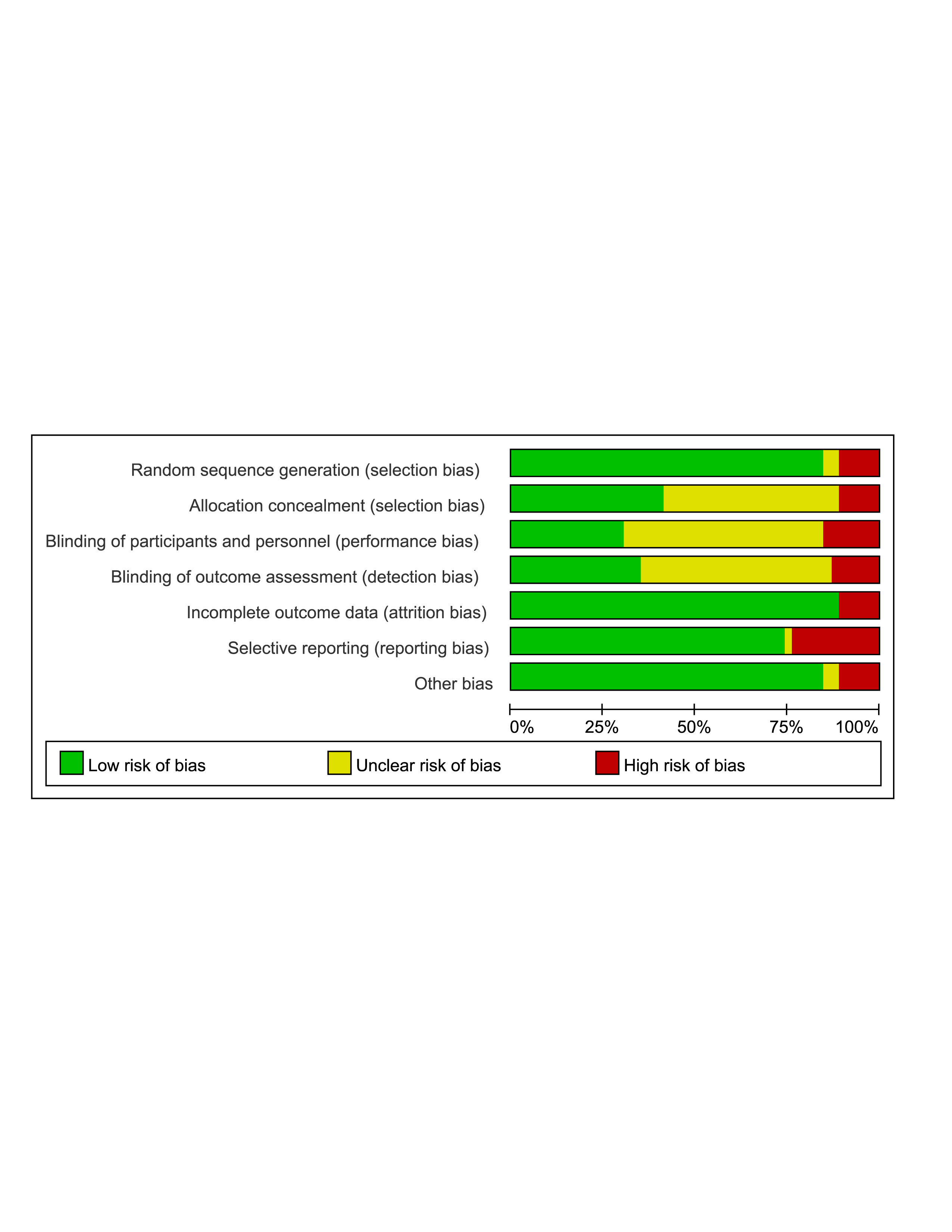


**Supplement Figure 9** Risk of bias graph


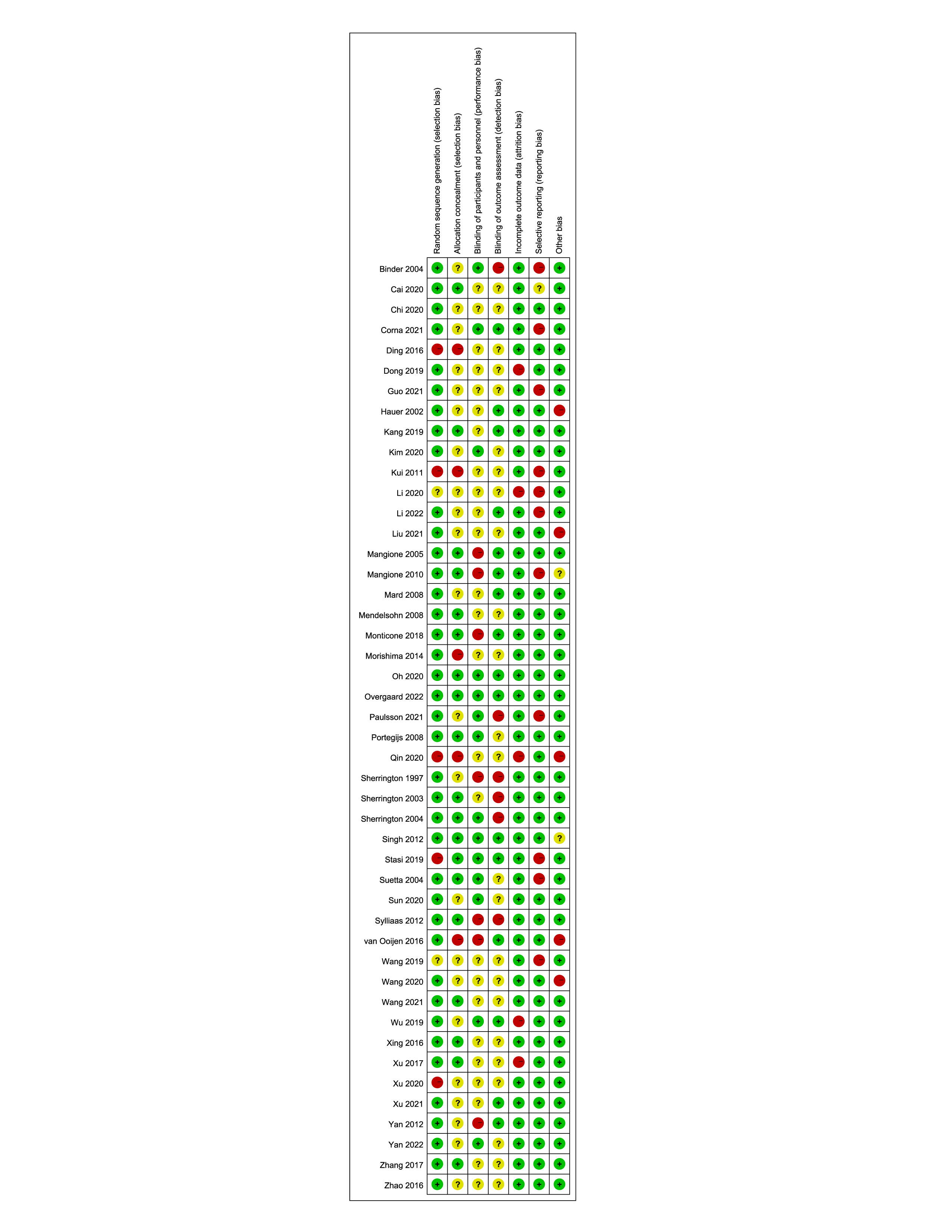


**Supplement Figure 10** Risk of bias summary

**Supplement Table 1** Relative effect sizes of different exercise interventions’ efficacy based on ADL


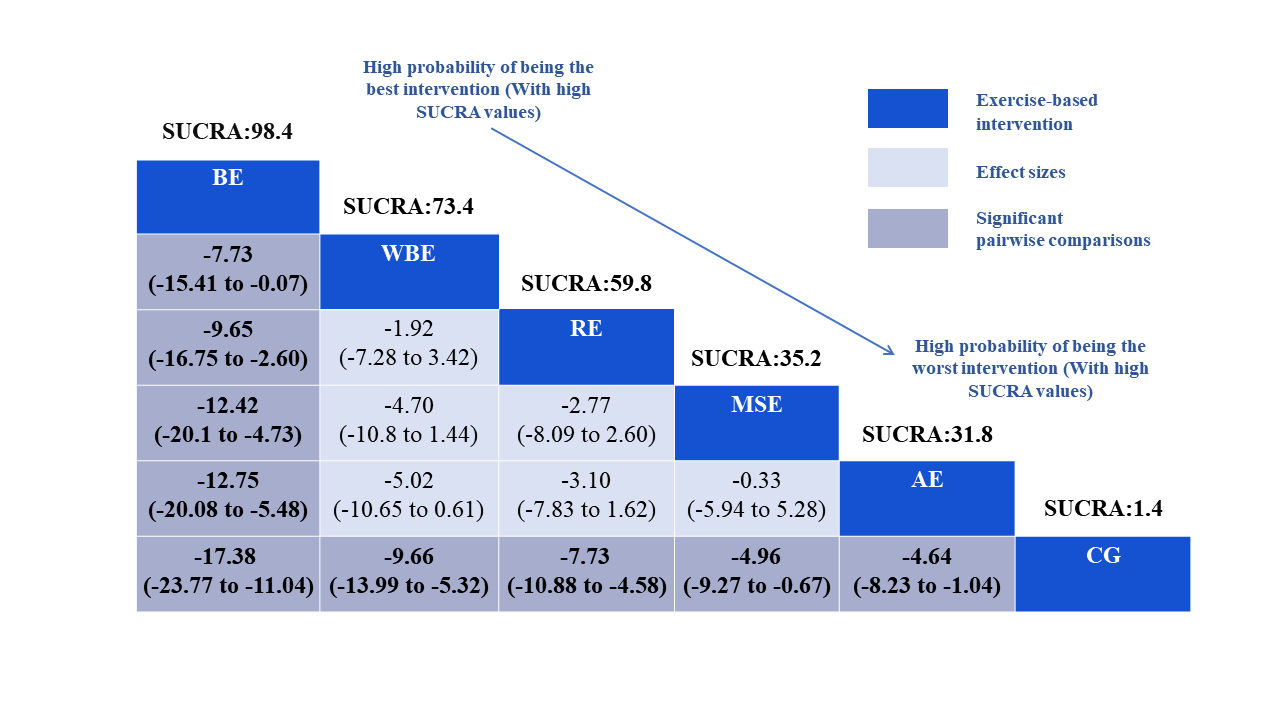


Comparative effectiveness results for ADL. Each cell shows an MD with a 95%CrI. Some numbers at the top of boxes are SUCRA values. The dark blue boxes indicate the type of campaign intervention, blue grey boxes represent significant pairwise comparisons were highlighted. AE, aerobic exercise; BE, balance exercise; CG, control group; Crl, credibility interval; MD, mean difference; MSE, muscle strength exercise; RE, resistance exercise; SUCRA, surface under cumulative ranking curve values; WBE, weight-bearing exercise.

**Supplement Table 2** Relative effect sizes of different exercise interventions’ efficacy based on walking capacity


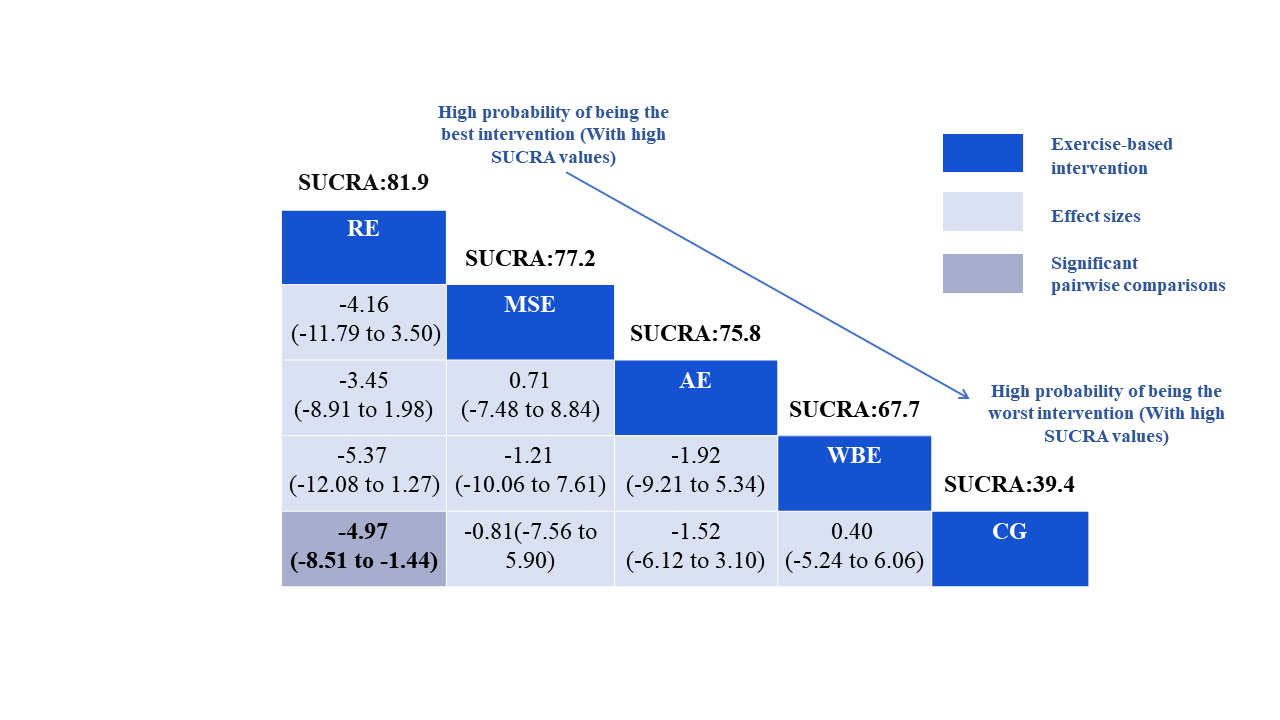


Comparative effectiveness results for walking capacity. Each cell shows an MD with a 95%CrI. Some numbers at the top of boxes are SUCRA values. The dark blue boxes indicate the type of campaign intervention, blue grey boxes represent significant pairwise comparisons were highlighted. AE, aerobic exercise; CG, control group; Crl, credibility interval; MD, mean difference; MSE, muscle strength exercise; RE, resistance exercise; SUCRA, surface under cumulative ranking curve values; WBE, weight-bearing exercise.

**Supplement Table 3** Relative effect sizes of different exercise interventions’ efficacy based on balance ability


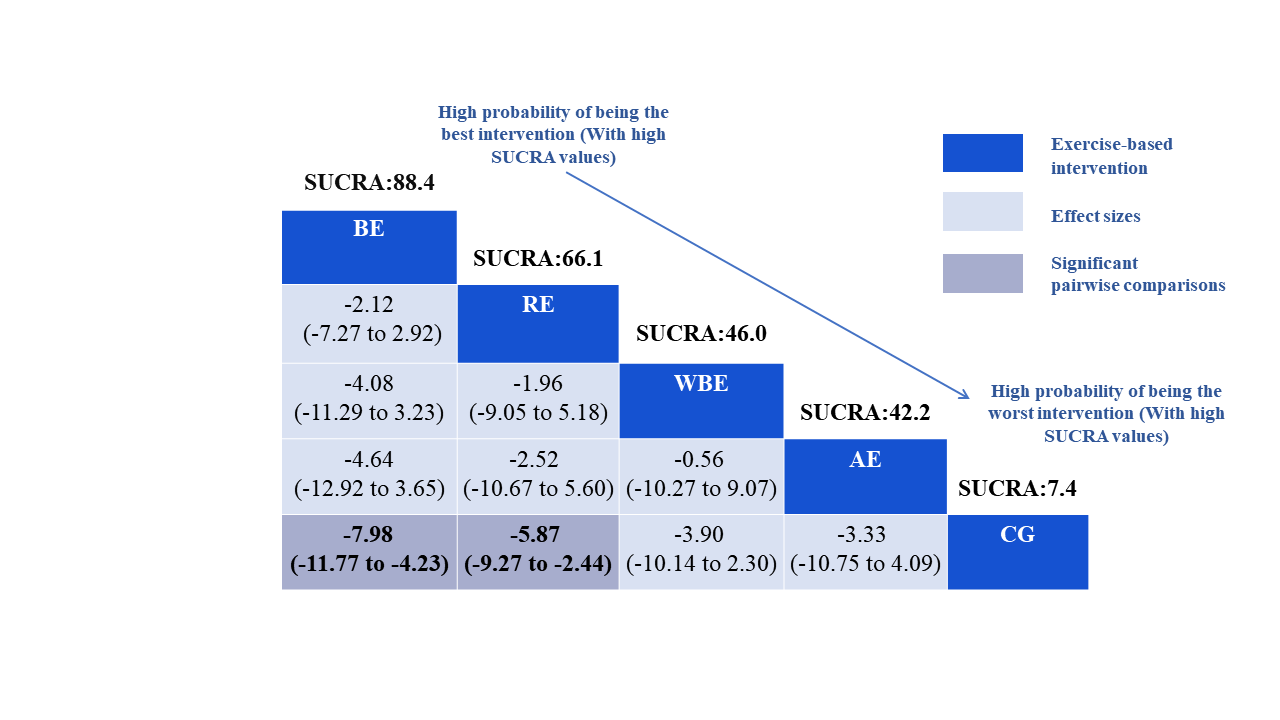


Comparative effectiveness results for balance ability. Each cell shows an MD with a 95%CrI. Some numbers at the top of boxes are SUCRA values. The dark blue boxes indicate the type of campaign intervention, blue grey boxes represent significant pairwise comparisons were highlighted. AE, aerobic exercise; BE, balance exercise; CG, control group; Crl, credibility interval; MD, mean difference; RE, resistance exercise; SUCRA, surface under cumulative ranking curve values; WBE, weight-bearing exercise.
